# Supplementary material for: Reprogrammable, intelligent soft origami LEGO coupling actuation, computation, and sensing
Source: Innovation (Camb). 2023 Nov 29;5(1):100549. doi: 10.1016/j.xinn.2023.100549 (PMC10772819; doi:10.1016/j.xinn.2023.100549)
Supplement: Document S1. Figures S1–S23 and Table S1 [file mmc1.pdf]

The Innovation, Volume *5*

Supplemental Information

Reprogrammable, intelligent soft origami LEGO coupling actuation, computation, and sensing

Zhongdong Jiao, Zhenhan Hu, Yuhao Shi, Kaichen Xu, Fangye Lin, Pingan Zhu, Wei Tang, Yiding Zhong, Huayong Yang, and Jun Zou

The Innovation, Volume [redacted]

Supplemental Information

Reprogrammable, intelligent soft origami LEGO coupling actuation, computation, and sensing

Zhongdong Jiao, Zhenhan Hu, Yuhao Shi, Kaichen Xu, Fangye Lin, Pingan Zhu, Wei Tang, Yiding Zhong, Huayong Yang, and Jun Zou

Fabrication of the ReISOs

The ReISO can be readily fabricated through elastomer casting or LCD 3D printing. In the elastomer casting, all the molds are made of polylactic acid (PLA) and manufactured with a 3D printer (Trianglelab, Dforce 300). We used two kinds of elastomers: the chamber of the soft origami is made of E630 elastomer (Shenzhen Hong Ye Jie Technology Co., Ltd.), and the male and female connectors are made of E650 elastomer (Shenzhen Hong Ye Jie Technology Co., Ltd.). As illustrated in Figure S1, the fabrication process includes four steps: (1) Two components of elastomers and pigment were mixed with a glass rod and degassed in a vacuum container. (2) The elastomer mixtures were then poured into the molds and heated in an oven (DZF-6090AB, Lichen) at 65 °C for 30 min. (3) The cured elastomers were removed from the molds. (4) The elastomer components were stuck together using silicone adhesive (CT772B, Odake). In the LCD 3D printing, the ReISO is fabricated by curing flexible resin in a commercial 3D printer (LD-002H, CREALITY-3D), as shown in Figure S2. Then the control tube and intelligent tube are glued to the cured resin. The dimensions of the ReISOs are depicted in Figure S19.

The control system for the ReISOs

The ReISOs in the logic characterization experiments and the soft logic circuits are controlled by a custom-built pneumatic control system shown in Figure S13. The vacuum pressure is generated by a vacuum pump (V-I240SV pump, VALUE) and regulated with a pressure regulator (ITV 2090, SMC). The control signals for the pressure regulator are generated by a signal generator. The solenoid valve (KVE32PL24FF valve, Kamoer) is used to fold/unfold the ReISOs. The two inlet ports and the outlet port are connected to the vacuum, atmospheric air, and soft origami, respectively. The operation states of the solenoid valve are controlled via the digital signals produced by an Arduino board (MEGA2560 R3) and relays. The pressures at port “A” and port “Q” are measured using a custom-built data acquisition system. The

internal diameter of the elastomer tubes used in the control system is 2 mm.

Finite element model for the soft origami

To guarantee reliable blocking behavior, the contraction of the soft origami must be larger than the contraction required to block the airflow completely. We investigated the influence of the structure parameters on the deformation performances of the soft origami by finite element method (FEM). The commercial package ABAQUS 2019 was used for the finite element analyses. The soft origami was modeled with a Yeoh hyperelastic model, where material parameters were determined experimentally by uniaxial tensile tests. In Figure S10, we report the numerically predicted deformation performances of soft origami as a function of the initial origami height H_0 and side length L_s. Combined with the results in Figure 2A-C, we can obtain a design guideline of the soft origami. In this work, we choose H_0 = 30 mm and L_s = 30 mm to carry out characterization experiments and build soft machines.

Analytical model of the ReISO

The fluidic soft origami can be equivalent to an electric circuit, as shown in Figure 2F-G. In this fluidic circuit, the fluidic pressure (Pa), mass flow rate (kg/s), fluidic resistance (Pa • s/kg), and fluidic capacity (kg/Pa) are analogous to the voltage (V), current (A), resistance (Ω), and capacity (F) of electric circuit, respectively. R_{tube1}, R_{tube2}, and R_{capi} represent the fluidic resistance of the control tube, the upper part of the intelligent tube, and the capillary tube, respectively. C_{cham} and C_Q denote the fluidic capacities of the chamber of the soft origami and the airtight channel that connects with port "Q", respectively. P_{cham} and P_Q are the pressure of the chamber and the airtight channel, respectively. S_A is a mechanical switch that represents the state of input "A", and S_K is a fluidic relay that represents the state of the intelligent tube. When P_{cham} > P_{kink}, the relay SK is in state 1 (Figure 2F) where port "Q" is subjected to the vacuum. When $P_{cham} \leqslant P_{kink}$, the relay SK switches to state 2 (Figure 2G) where port "Q" is

connected to the atmosphere.

The Reynolds number in the system is approximately 10-100, which is significantly smaller than the critical Reynolds number (Re ~ 2,300) for the transition to turbulent flow. Then the fluidic resistance R_{fluid} can be calculated according to the Darcy-Weisbach equation for laminar flow:

$$R_{fluid} = \frac{\Delta P}{\dot{m}} = \frac{128\mu L}{\pi\rho D^4} \tag{1}$$

where ΔP is the pressure difference between the two ends of the fluidic tube, $\dot{m}$ is the mass flow rate of air, μ is the dynamic viscosity of air, ρ is the density of air, D is the internal diameter of the tube, and L is the length of the tube.

The fluidic capacity can be calculated with the ideal gas equation of state:

$$C_{fluid} = \frac{dm}{dP} = \frac{VM}{RT} \tag{2}$$

where V is the volume of the chamber or tube, M is the molar mass of air, R is the universal gas constant, and T is the temperature.

In the folding process, the air flows from the chamber to the vacuum source via R_{tube1}. When the pressure of the chamber P_{cham} reaches P_{kink} (the kinking pressure of the ReISO), the air flows from the atmosphere to the airtight channel that connects with port "Q" via R_{capi}. The pressures of the chamber and the intelligent tube can be described as:

$$P_S = C_{cham}\frac{dP_{cham}}{dt}R_{tube1} + P_{cham} \tag{3}$$

$$R_{capi}C_Q\frac{dP_Q}{dt} + P_Q = 0 \tag{4}$$

With the condition $t = 0$, $P_{cham} = P_{atm}$; $t = t_{kink}$, $P_Q = P_{Q0}$, the solution to Eq. 4-5 is:

$$P_{cham} = \frac{P_S}{R_{tube1}C_{cham}} + e^{-\frac{1}{R_{tube1}C_{cham}}t}\left(P_{atm} - \frac{P_S}{R_{tube1}C_{cham}}\right) \tag{5}$$

$$P_Q = \begin{cases} P_{Q0} & t < t_{kink} \\ P_{Q0}e^{-\frac{1}{R_{capi}C_Q}(t-t_{kink})} & t \geq t_{kink} \end{cases} \tag{6}$$

$$P_{Q0} = \frac{R_{capi}}{R_{tube2} + R_{capi}} P_S \tag{7}$$

$$t_{kink} = -R_{tube1} C_{cham} \ln \frac{R_{tube1} C_{cham} P_{kink} - P_S}{R_{tube1} C_{cham} P_{atm} - P_S} \tag{8}$$

where P_{Q0} is the pressure of port "Q" in the unfolding state.

In the unfolding process, the air flows into the chamber via R_{tube1}. When the internal pressure of the chamber is higher than P_{kink}, the intelligent tube is open and the air at the "Q" port is removed.

$$R_{tube1} C_{cham} \frac{dP_{cham}}{dt} + P_{cham} = 0 \tag{9}$$

$$\left(\frac{P_Q}{R_{capi}} + C_Q \frac{dP_Q}{dt} \right) R_{tube2} + P_Q = P_S \tag{10}$$

With the condition $t = 0$, $P_{cham} = P_S$; $t = t_{open}$, $P_Q = P_{atm}$, the solution to Eq. 10-11 is:

$$P_{cham} = P_S e^{-\frac{1}{R_{tube1} C_{cham}} t} \tag{11}$$

$$P_Q = \begin{cases} P_{atm} & t < t_{open} \\ X_2 + e^{-X_1 \left(t - t_{open}\right)} \left(P_{atm} - X_2\right) & t \geq t_{open} \end{cases} \tag{12}$$

$$X_1 = \frac{R_{tube2} + R_{capi}}{R_{tube2} R_{capi} C_Q}, \quad X_2 = \frac{P_S}{R_{tube2} C_Q}$$

$$t_{open} = -R_{tube1} C_{cham} \ln \frac{P_{open}}{P_S} \tag{13}$$

The logic response time (the time required to switch from Q = "1" to Q = "0" or from Q = "0" to Q = "1") of the ReISO can be expressed as Eq. 15 and Eq. 17

$$t_{1 \to 0} = -R_{capi} C_Q \ln \frac{P_{Q1}}{P_{Q0}} + t_{kink} \tag{14}$$

$$P_{Q1} = \left(P_{Q0} - P_{atm}\right) \times 10\% + P_{atm} \tag{15}$$

$$t_{0 \to 1} = -\frac{1}{X_1} \ln \frac{P_{Q2} - X_2}{P_{atm} - X_2} + t_{open} \tag{16}$$

$$P_{Q2} = \left(P_{Q0} - P_{atm}\right) \times 90\% + P_{atm} \tag{17}$$

The state that $P_Q \geqslant P_{Q1}$ is defined as the logic low state, and the state that $P_Q \leqslant P_{Q2}$ is defined as the logic high state (here the fluidic signals processed by the ReISO are vacuum pressures, thus the magnitude of P_Q, P_{Q1}, and P_{Q2} is negative).

For the ReISOs in this work, we choose the elastomer tubes with an internal diameter of 2.0 mm and an external diameter of 3.0 mm as the input tubes and intelligent tubes owing to their superior kinking properties. The tubes with this dimension allow the ReISOs to exhibit a relatively fast response (~ 0.2 s), thus, $t_{0\to1}$ is very small (< 1 s). In contrast, the capillary tubes have high fluidic resistance and can be utilized to modulate the response characterization of the ReISOs. Then we employed this analytical model to calculate the $t_{1\to0}$ of the ReISOs. The results are shown in Figure 2I, in which the calculated response time agrees well with the experimental values. The agreement between the model and experiment illustrates the potential of analyzing complex soft circuits constructed with ReISOs.

The frequency characteristic and fatigue test of the ReISOs

We further studied the logic performances of ReISO under frequencies between 0.1 and 2.5 Hz. The highest frequency that allowed for the logic operation was 2.5 Hz (Figure S8F). The result of a continuous fatigue test of the soft origami sample over 10,000 operating cycles under a pressure of -80 kPa and a frequency of 2.5 Hz is shown in Figure S9. The soft origami consistently exhibited the same output pressure and logic operation capability.

The working principle of the soft turtle

As the soft turtle swims in the tank, its legs swing periodically in the water (Figure 5D). The swinging movement makes the legs be subjected to a reaction torque, which can be calculated with the drag equation.

$$F_d = \frac{1}{2}\rho v^2 C_d A \tag{18}$$

$$M_d = F_d L = \frac{1}{2}\rho v^2 C_d A L_d \tag{19}$$

where F_d is the drag force, ρ is the density of water, v is the moving velocity of the legs relative to the water, C_d is the drag coefficient, A is the projected area of the legs in the moving direction, L_d is the equivalent distance between the turtle leg and the axis of the soft origami, M_d is the drag torque.

If the swinging speeds are the same in the folding and unfolding processes, the turtle will stay put. However, for the ring oscillator, the soft origami is folded via the intelligent tube whose internal diameter is 2 mm, and unfolded via the capillary tube whose internal diameter is 0.7 mm. The differences in the internal diameter lead to different flow rates, therefore causing the morphing speed differences between folding and unfolding processes (Figure 5D). As a result, the turtle moves toward the thrust direction of the folding process.

$$W = \int_{\theta_1}^{\theta_2} M_d d\theta = \frac{1}{2}\rho v^2 C_d A L_d d\theta \tag{20}$$

$$v_{fold} > v_{unfold} \tag{21}$$

$$W_{fold} > W_{unfold} \tag{22}$$

where W is the amount of the work, W_{fold} and W_{unfold} are the amounts of the work in the folding and unfolding processes, respectively. v_{fold} and v_{unfold} are the moving velocity of the legs in the folding and unfolding processes, respectively. θ is the twisting angle of the leg.

The swimming speed of the soft turtle can be modulated by tuning the oscillation frequency and range (twisting angle) of the soft ring oscillator. These properties are determined by two key parameters: the actuation pressure and the internal diameter of the capillary tube. As illustrated in Figures S18A and S18C, an increase in the internal diameter of the capillary tube results in higher oscillation frequency, while having a negligible influence on the oscillation range. Conversely, the oscillation range first

increases with the decrease in vacuum pressure, but then remains at a certain level (actuation pressure < -40 kPa). The oscillation frequency has slight variations with varying vacuum pressure (Figures S18B and S18D).

Characterization experiments of the ReISOs

The experimental setup for actuation performance tests is demonstrated in Figure S11. Two high-resolution cameras are placed on top of the module and in front of the module, respectively. The angle and height variations of the module are recorded by the camera at a frame rate of 30 fps.

The setup for kinking characterization of the elastomer tubes is illustrated in Figure S12. One end of the tube is fixed, while the other end of the tube is driven by two stepping motors. Stepping motor 1 is responsible for twisting the tube to a certain angle, and stepping motor 2 is used to compress the tube.

The working principle of the soft ring oscillator

When the source ports of the three ReISOs were connected to a constant vacuum pressure, module "A" was actuated first (the module that is actuated first is random). As described in Figure S15A, the deformation of module "A" blocked the input port of module "B" in S1. Subsequently, module "C" was evacuated via the output port of module "B". When module "C" became the logic high state (the internal intelligent tube was kinked), the ring oscillator came into state S2 (Figure S15B), with the input port of module "A" being blocked and module "A" returning to its original shape. The opening of the airflow in module "A" made module "B" be subjected to vacuum pressure. When the internal pressure of module "B" reached the kinking pressure, the input port of module "C" is blocked and module "C" returned to the logic low state gradually (state S3, Figure S15C). The reopening of the airflow of module "C" initiated the actuation of module "A", and the soft ring oscillator entered the next cycle.

The JK flip flop and D flip flop circuits enabled by the ReISOs

JK Flip Flop

The JK flip flop is a gated SR latch with the addition of a clock input that prevents the illegal or invalid output when both inputs “S” and “R” are set to “1” (Figure S16A-i). In this circuit, the inputs “S” and “R” are labeled as “J” and “K”, respectively. The two input ports are replaced by two 3-input NAND gates with the second input of each gate connected to a clock input and the third input connected to the output “Q” or “$\overline{Q}$”. This feedback enables only one of its two input ports, either SET or RESET to be active at any time under normal switching. Therefore, the invalid condition of “J” = “1” and “K” = “1” state can be used to produce a “toggle action” as the two inputs are now interlocked.

In this work, ten ReISOs were employed to construct a soft JK flip flop circuit, which is positive edge-triggered, as depicted in Figure S16A-ii. When both inputs “J” and “K” were “0”, the outputs held the previous state. If “J” was set to “1” and “K” was set to “0”, the rising edge of the clock input caused the output “Q” to become “1” and “$\overline{Q}$” to become “0” (Figure S16A-iii-iv and Movie S8). Similarly, when “J” was set to “0” and “K” was set to “1”, the rising edge of the clock input caused the output “Q” to become “0” and “$\overline{Q}$” to become “1”. When both “J” and “K” were set to “1”, the output “Q” and “$\overline{Q}$” toggled the previous state with each rising edge of the clock input.

D Flip Flop

The D flip flop (delay flip flop) is designed by adding a NOT gate between the inputs “S” and “R” of a gated SR latch (Figure S16B-i). Then inputs “S” and “R” become complements of each other and are never equal to each other at the same time, allowing us to control the toggle action of the flip flop using one single input. The D flip flop has two inputs: the data input “D” and the clock input “Clk”. In this circuit, the data input is delayed up to one clock pulse before it is displayed in the output. Therefore, the D flip flop can be used to store data at a predetermined time and hold it until it is needed.

As illustrated in Figure S16B-ii, a soft D flip flop was created using 9 ReISOs. When the clock input was set to “0”, the outputs “Q” and “$\overline{Q}$” were held in the previous state. In contrast, the high level of the clock input enabled the input “D” to be copied to the output “Q” (Figure S16B-iii-iv and Movie S8).

The soft frequency enabled by the ReISOs

A soft frequency divider is based on a D flip flop circuit with the output "$\overline{Q}$" connected directly back to the data input "D" (Figure 4G-i). This feedback ensures the output pulses at "Q" have a frequency that is exactly one half of the input clock frequency. As illustrated in Figure 4G-ii, the soft frequency divider was created using 9 ReISOs. It can be seen that the output "Q" only changes state on the rising edge of the pulse clock stream "Clk" (Figure 4G-iii-iv). Each rising edge occurs once every cycle, but the output "Q" requires two changes to complete a cycle. Therefore, the output "Q" changes at half the rate of the pulse clock stream "Clk". In other words, the clock frequency was divided by two. The frequency can be further divided by cascading more D flip flops. This demonstration illustrates that the ReISOs are able to process fluidic signals.

The vacuum leakage rate of the ReISOs

The vacuum leakage has some influences on the pressure magnitude of the vacuum system. As demonstrated in Figure S21, the vacuum leakage rate increases with the increase of the internal diameter of the capillary tube and the quantity of the ReISOs. When eight ReISOs with a capillary tube of 0.5 mm are connected in parallel, the vacuum system can retain 80.4% of the initial vacuum pressure (-56.3 kPa), which is sufficient to power soft actuators.

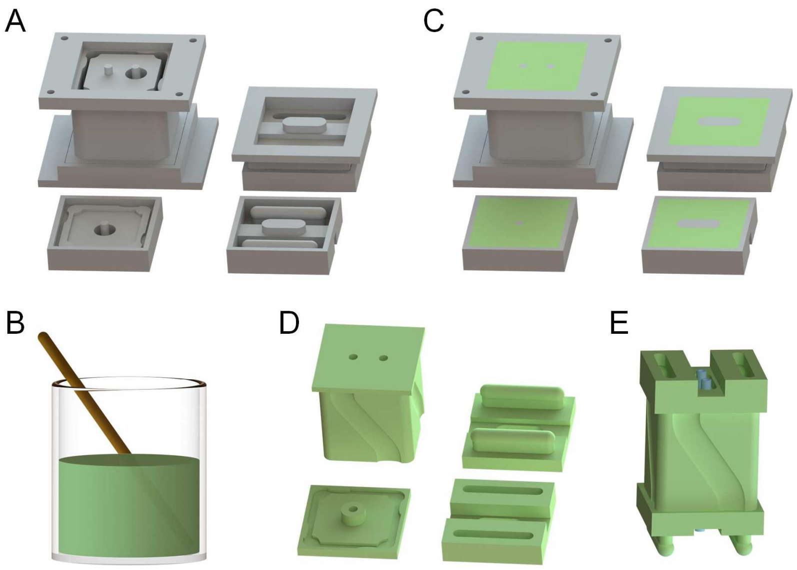

Figure S1. The ReISO is fabricated by elastomer casting. a All the molds are manufactured with a 3D printer. **b** The elastomer and pigment are mixed, stirred with a glass rod, and degassed in a vacuum container. **c** The liquid elastomer is poured into the mold and heated in an oven. **d** The cured elastomers are removed from the molds. **e** All the parts are stuck together using silicone adhesive, forming a soft origami.

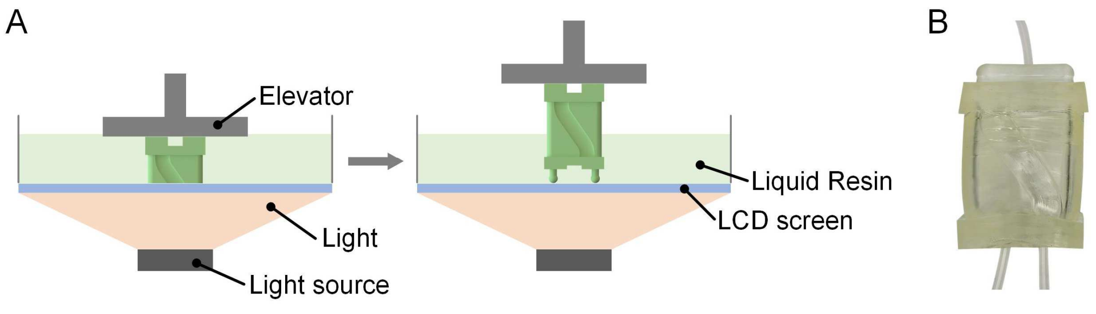

Figure S2. The ReISO is fabricated with an LCD printer. (A) The printing process of the ReISO. (B) The ReISO made of flexible resin.

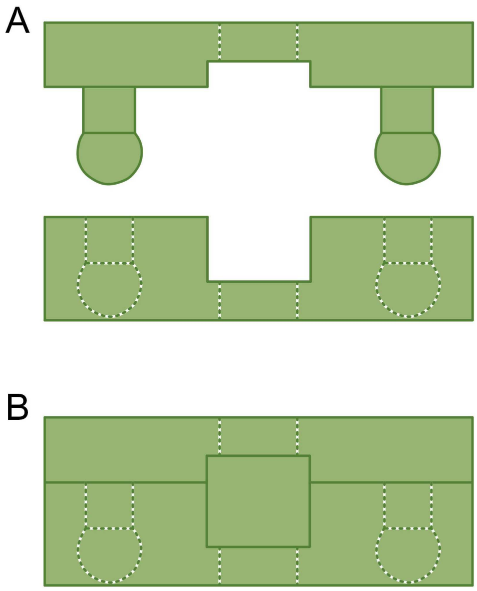

Figure S3. The plug-and-play connectors of the ReISOs. (A) Before assembly. (B) After assembly.

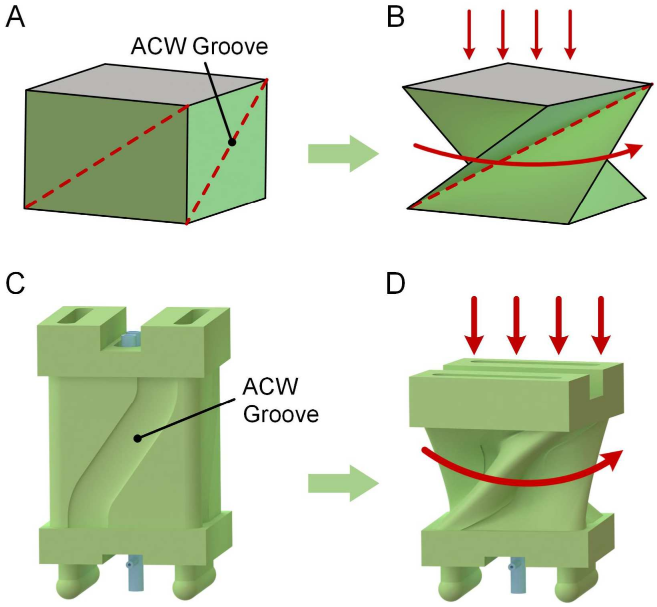

Figure S4. Schematic illustration of the Kresling origami and ReISO with anticlockwise grooves. (A-B) The anticlockwise Kresling origami is in unfolded (A) and folded (B) states. The red dashed lines represent the creases. The red arrows indicate the folding direction of the Kresling origami. ACW represents anticlockwise. (C-D) Schematic illustration of the anticlockwise ReISO in unfolded (C) and folded (D) states. The red arrows indicate the deformation direction of the soft origami. The grooves in the sides are the creases of the soft origami.

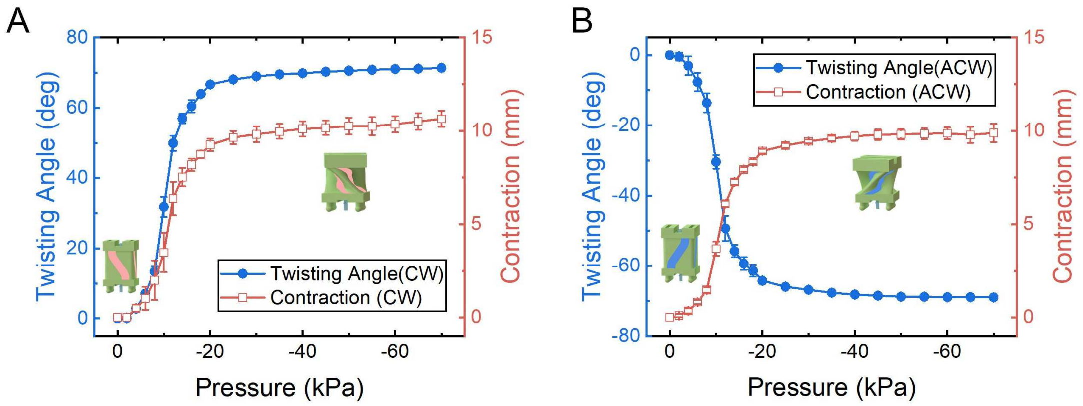

Figure S5. The twisting angle and contraction of the ReISOs with clockwise grooves (A) and anticlockwise grooves (B) at different vacuum pressures. CW represents clockwise, ACW represents anticlockwise.

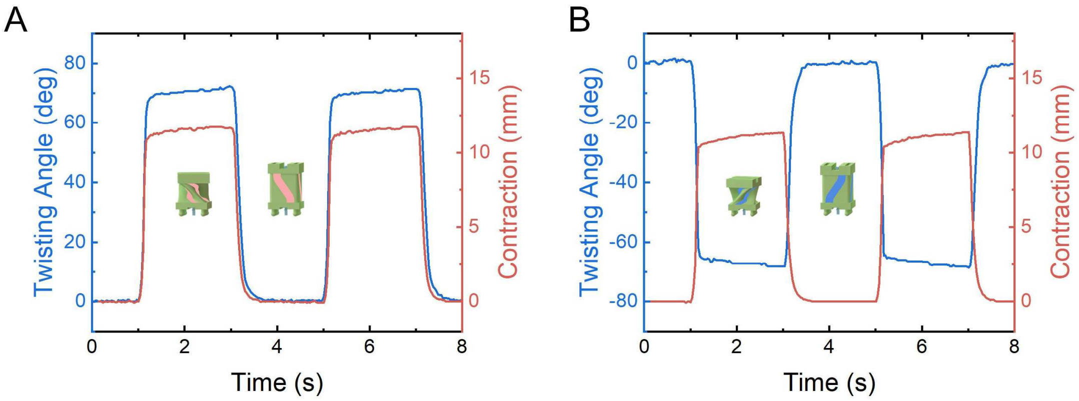

Figure S6. The dynamic deformation response of the ReISOs with clockwise grooves (A) and anticlockwise grooves (B).

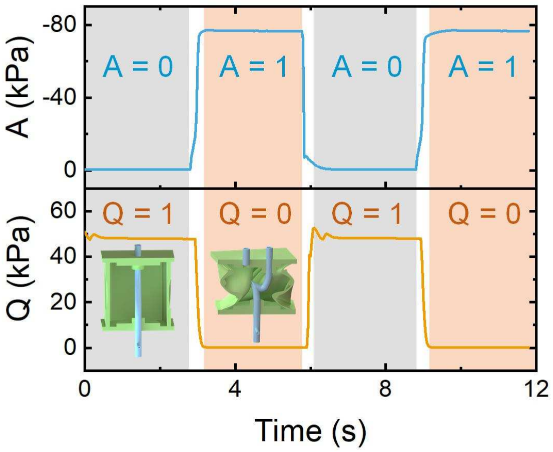

Figure S7. The pressure response of the ReISO when the "S" port is connected to a constant positive pressure of 60 kPa. In this case, the positive and vacuum pressure are defined as "1".

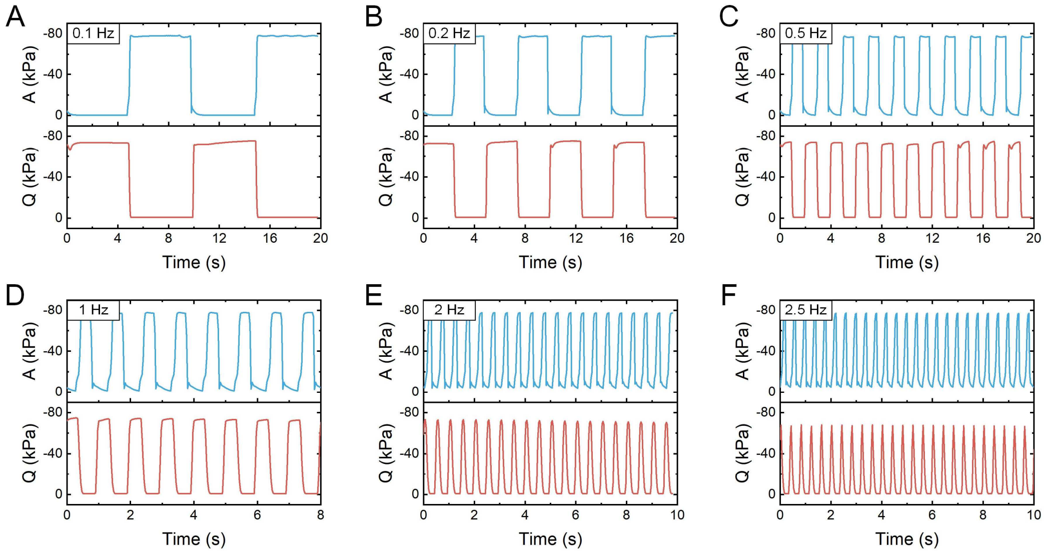

Figure S8. The pressure responses of the ReISO operating at different frequencies. (A) 0.1 Hz. (B) 0.2 Hz. (C) 0.5 Hz. (D) 1 Hz. (E) 2 Hz. (F) 2.5 Hz. The operation pressure is -80 kPa.

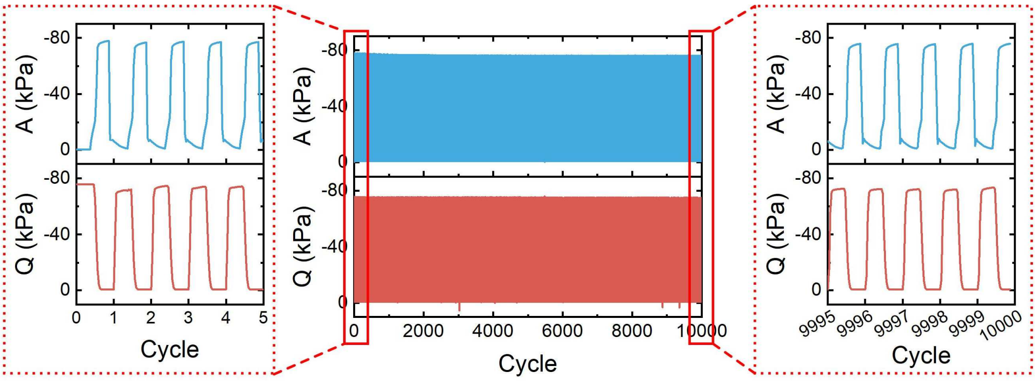

Figure S9. Fatigue test of the ReISO. A ReISO is actuated 10,000 cycles at a frequency of 2.5 Hz and a pressure of -80 kPa. The pressure response in the actuating process of the No. 1-5 and No. 9995-10000 cycles evidences the high durability of the ReISOs.

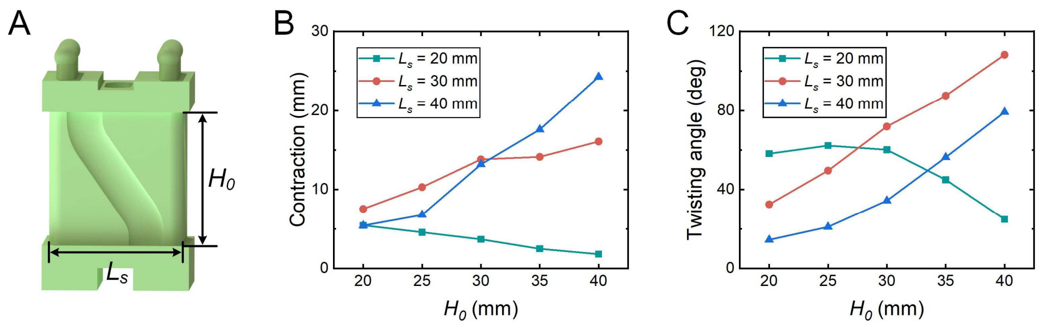

Figure S10. Finite element analysis of the soft origami with different dimensions. (A) The dimension schematic of the soft origami. H_0 is the initial origami height, L_s is the side length. (B) The contraction of the soft origami with different initial heights and side lengths. (C) The twisting angle of the soft origami with different initial heights and side lengths.

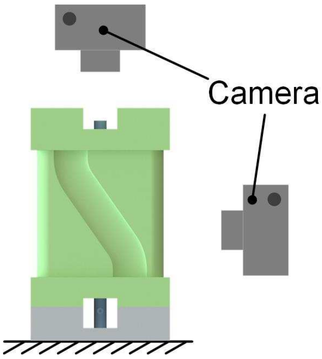

Figure S11. Experimental setup for the deformation measurement of the ReISOs.

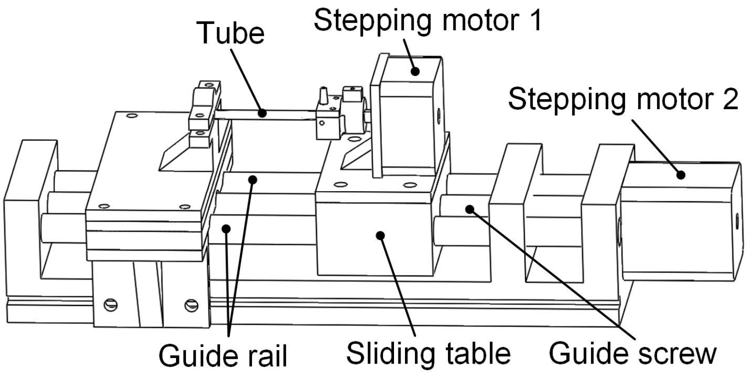

Figure S12. Experimental setup for the kinking characterization of the elastomer tubes.

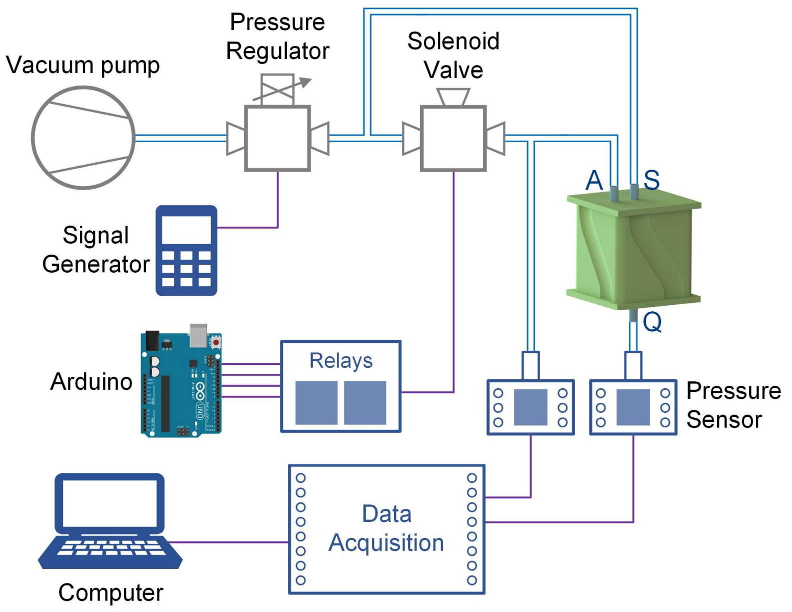

Figure S13. The schematic of the control system used to actuate the soft origami. The purple lines represent the control signals; the blue lines represent the fluidic channels.

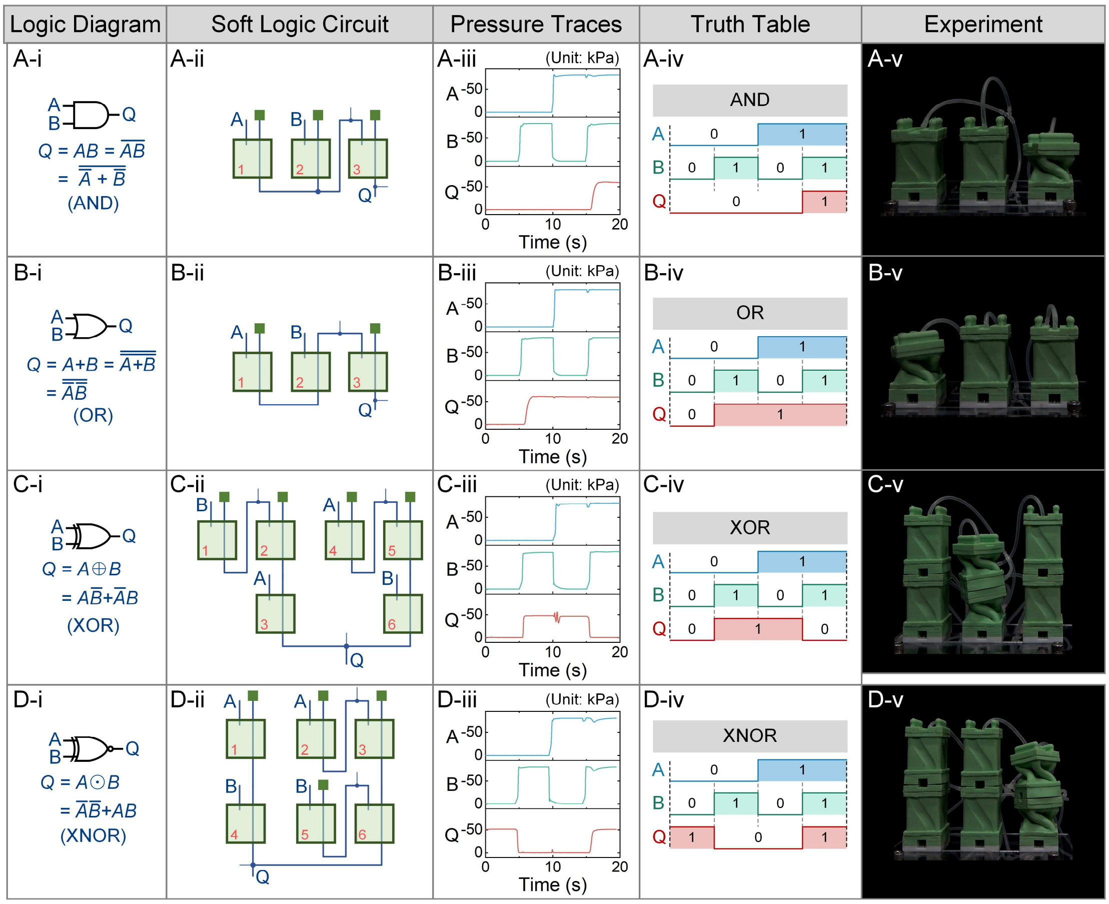

Figure S14. The reprogrammable combinatorial logic circuits based on the ReISOs. (A)-i The logic symbol and Boolean expression of the AND gate. (A)-ii The schematic of the soft AND gate circuit. (A)-iii The pressure traces of the soft AND gate. (A)-iv The truth table of the AND gate. (A)-v The experimental image of the soft AND gate. (B) The OR gate. (C) The XOR gate. (D) The XNOR gate. The green block represents the vacuum pressure.

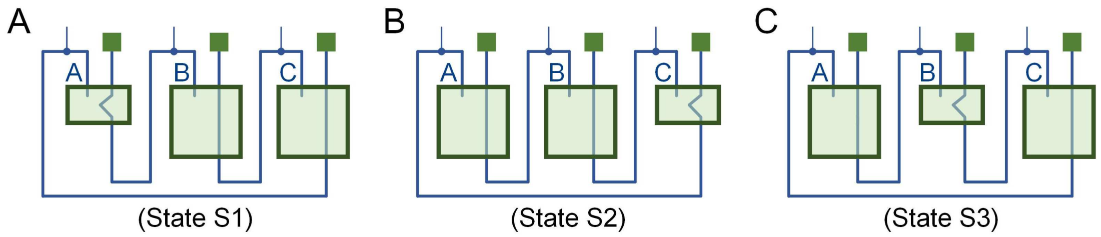

Figure S15. The oscillation sequences of the soft ring oscillator. Module “A” (A), module “C” (B), and module “B” (C) are folded sequentially.

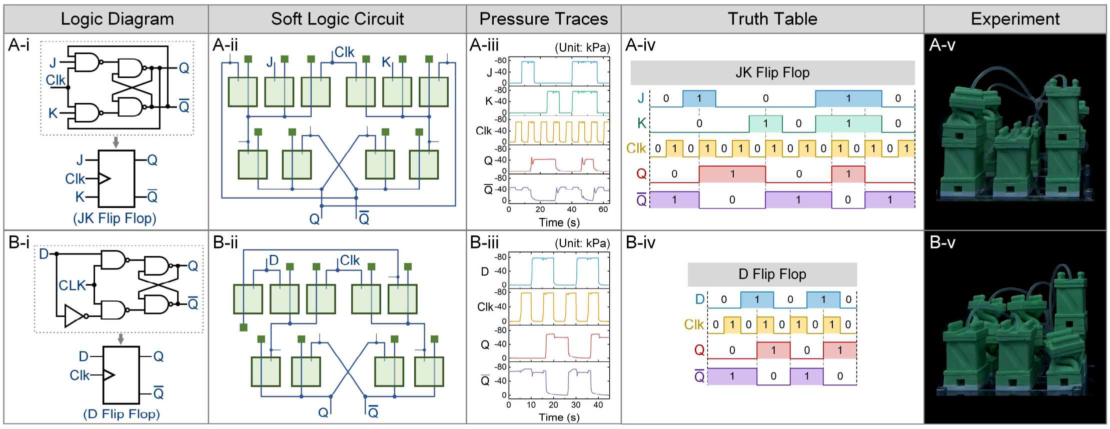

Figure S16. The reprogrammable sequential logic circuits based on the ReISOs. (A)-i The circuit diagram of the JK flip flop. (A)-ii The schematic of the soft JK flip flop circuit. (A)-iii The pressure traces of the soft JK flip flop. (A)-iv The truth table of the JK flip flop. (A)-v The experimental image of the soft JK flip flop. (B) The D flip flop. The green block represents the vacuum pressure.

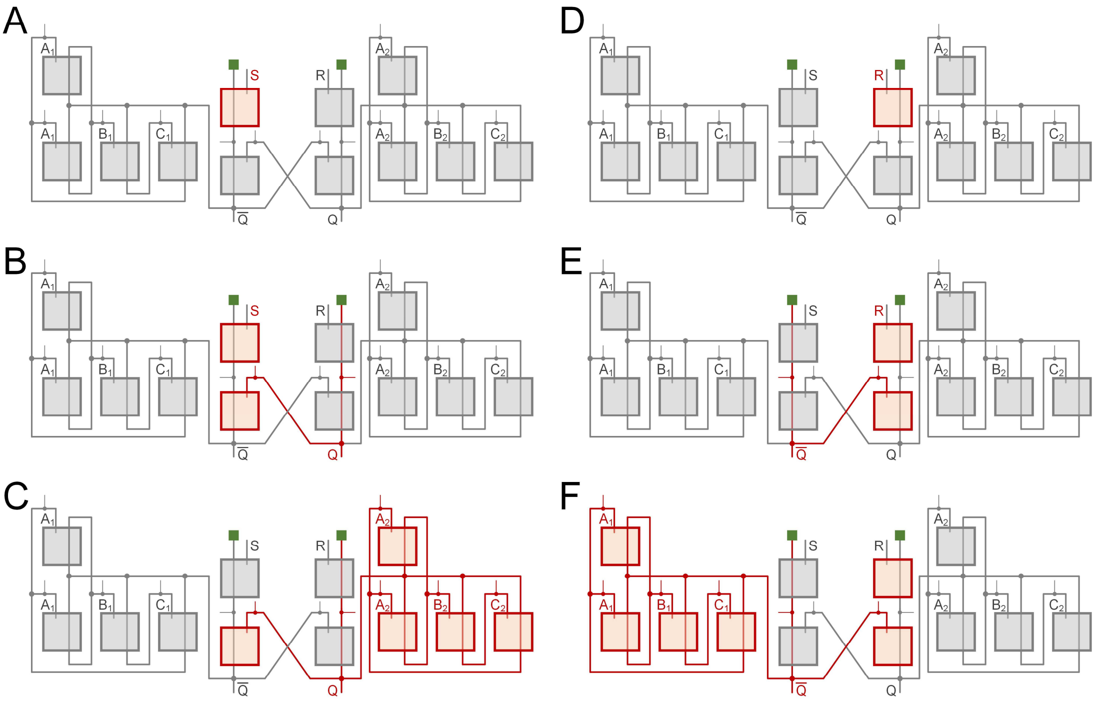

Figure S17. The control system of the untethered and autonomous soft turtle. (A) The twisting stimulus was applied to module "S". (B) The soft SR latch circuit detected this stimulus and stored the current state (Q = "1") in the circuit. (C) The Ring Oscillator-2 converted the constant pressure from port "Q" of the SR latch into oscillatory pressures, which drive the turtle to swim forward. The removal of the stimulus does not change the state of the SR latch circuit. (D) The twisting stimulus was applied to module "R". (E) The soft SR latch circuit detected this stimulus and stored the current state ($\overline{Q}$ = "1") in the circuit. (F) The Ring Oscillator-1 converted the constant pressure from port "$\overline{Q}$" of the SR latch into oscillatory pressures, which drive the turtle to swim backward.

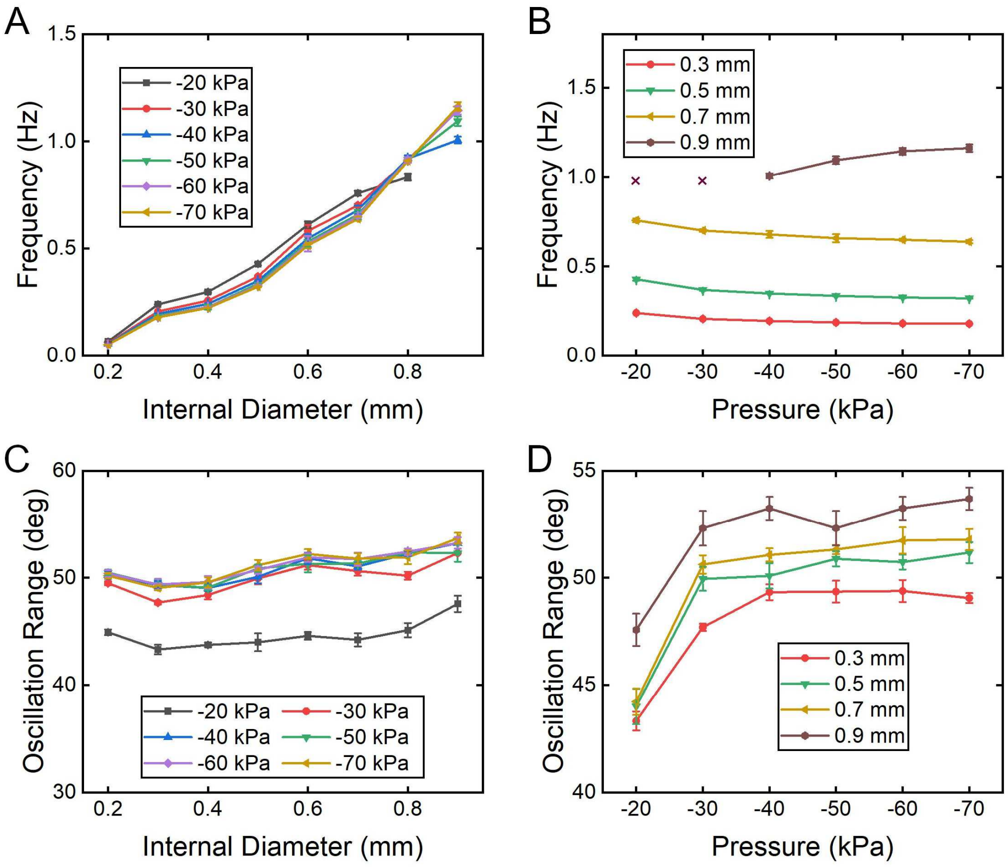

Figure S18. The oscillation frequency and range (twisting angle) can be adjusted by changing the inner diameter of the capillary tube and the actuation pressure of the ring oscillator. (A-B) Oscillation frequency as a function of the inner diameter of the capillary tube (A) and the actuation pressure (A). (C-D) Oscillation range as a function of the inner diameter of the capillary tube (C) and the actuation pressure (D).

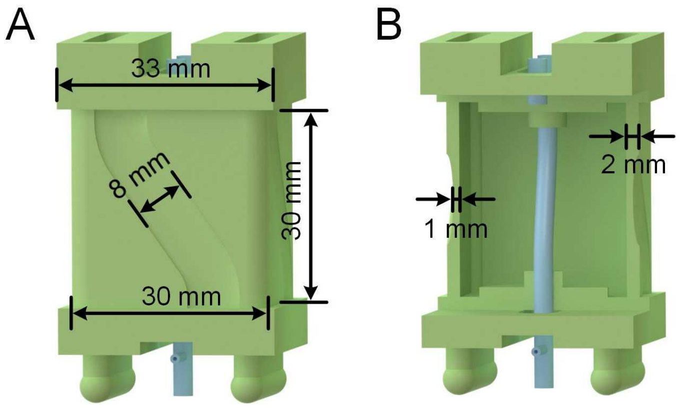

Figure S19. The dimension of the ReISO. (A) Front view. (B) cross-section view.

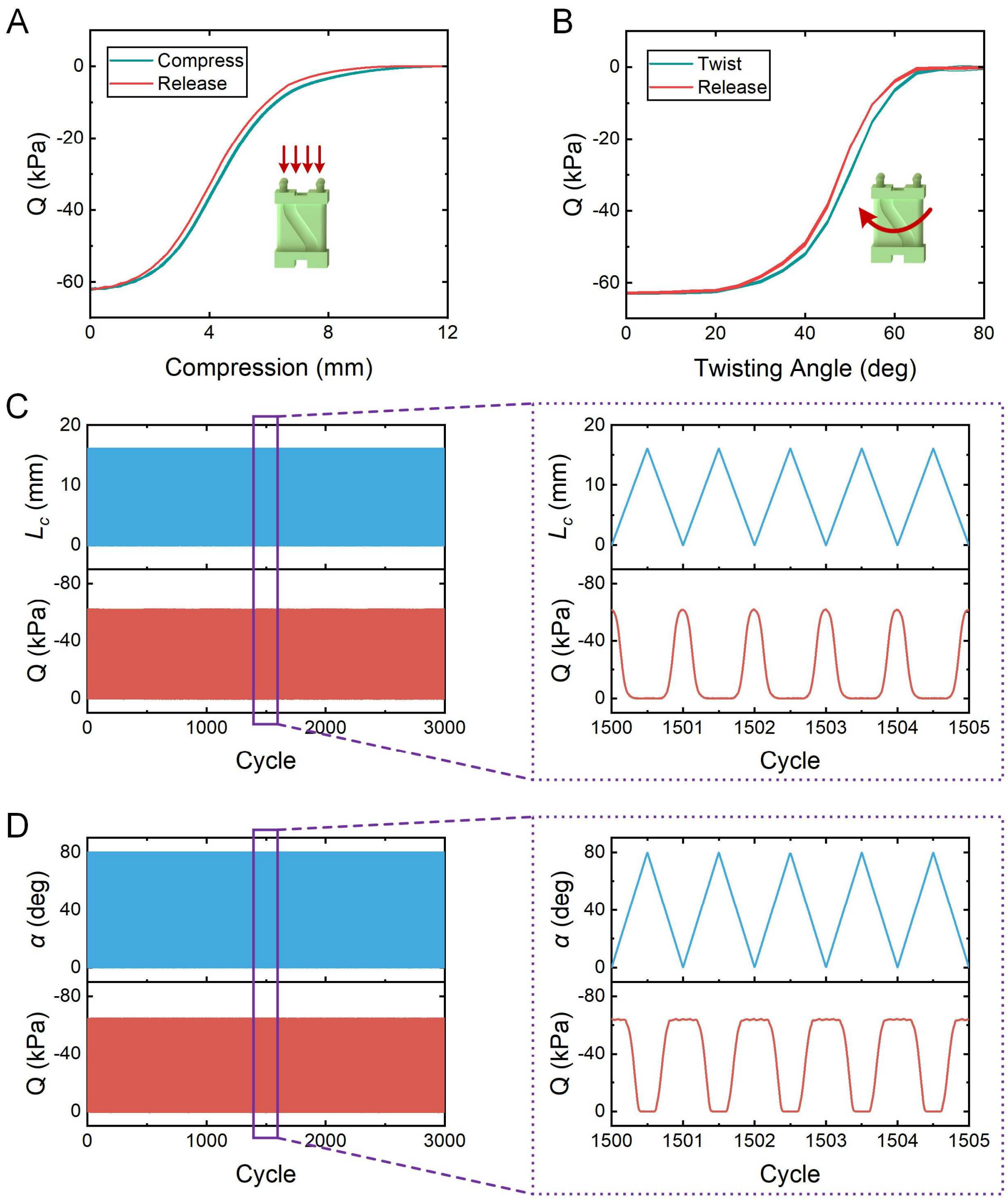

Figure S20. The sensing characteristic of the ReISOs. (A) The output pressure changes with varying compressions. The measurements were repeated five times. (B) The output pressure changes with varying twisting angles. (C) The fatigue test of the compression sensing capability of the ReISO. L_c represents compression. (D) The fatigue test of the twisting angle sensing capability of the ReISO. α represents twisting angle.

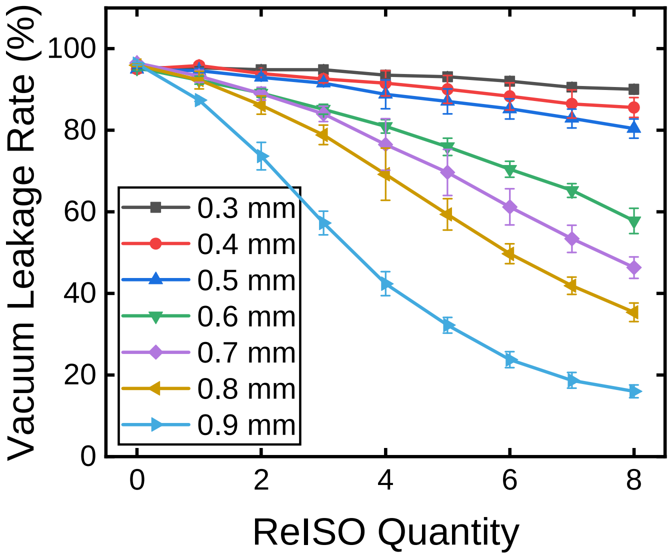

Figure S21. The vacuum leakage rate of the ReISOs with different capillary tube and module quantity.

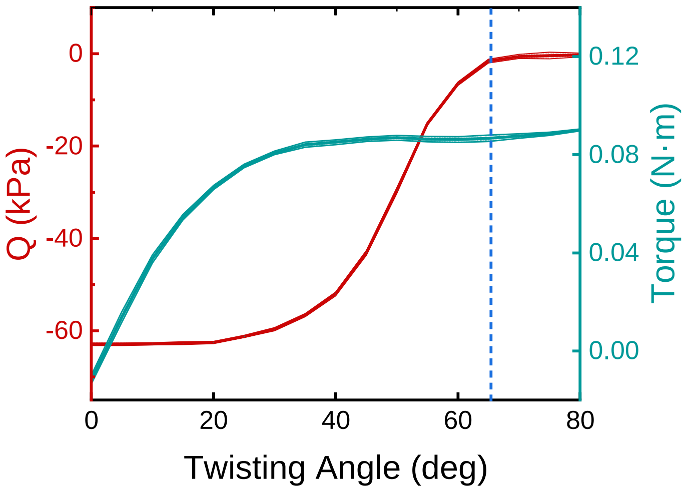

Figure S22. The torque and output pressure variations while twisting the ReISOs.

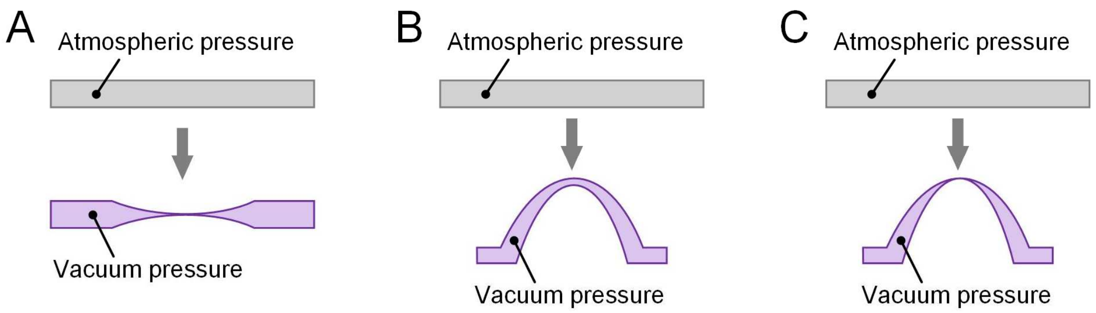

Figure S23. The deformation of the compressed elastomer tube. (A) The tube with a size of 1.5×2.0 mm collapses when subjected to vacuum pressure. (B) The tube with a size of 1.0×2.0 mm is difficult to be kinked due to smaller internal and external diameters. (C) The tube is successfully kinked.

Table S1. The Estimated cost of materials used for the fabrication of a ReISO.

Items	Amount	Unit	Cost
Elastomer	34	g	$ 0.693
Elastomer tube	0.2	m	$ 0.026
Silicone adhesive	2	mL	$ 0.133
Total cost			**$ 0.852**

Supplemental Movie Captions

Movie S1. The actuation capability of the ReISOs. The soft origami rotates clockwise and anticlockwise, respectively.

Movie S2. The logic operation capability of the ReISOs. Port “S” is connected to the pressurized air and vacuum, respectively. The vacuum/positive pressure and atmospheric pressure are defined as logic signal “1” and “0”, respectively.

Movie S3. The sensing capability of the ReISOs. The soft origami is able to detect twisting and pressing stimuli.

Movie S4. The damage-resistance of the ReISOs. The logic operation capability of the soft origami is not affected after being pricked with a needle.

Movie S5. The reconfigurable morphologies of the ReISOs. The ReISOs are configured into various morphologies to exhibit contraction movement, twisting movement, outward radial movement, inward radial movement, and bidirectional bending movement, respectively.

Movie S6. Reconfigurable soft robots based on the ReISOs. The soft rod-climbing robot is able to climb forward and backward along a metal rod. The soft manipulator is utilized to place triangular and square objects into holes with specific shapes.

Movie S7. Reprogrammable soft combinatorial logic circuits. The ReISOs are utilized to build fundamental combinatorial logic circuits, including the NOT gate, Buffer gate, NAND gate, NOR gate, AND gate, OR gate, XOR gate, and XNOR gate.

Movie S8. Reprogrammable soft sequential logic circuits. The ReISOs can be used to construct sequential logic circuits, including the ring oscillator, SR latch, JK flip flop, and D flip flop circuits.

Movie S9. Reprogrammable soft functional circuits. A soft full adder circuit and a soft frequency divider circuit are built by assembling the ReISOs.

Movie S10. The movements of an untethered soft turtle. The soft turtle is able to swim forward, swim backward, rotate clockwise, and rotate anticlockwise, respectively.

Movie S11. An untethered autonomous soft turtle that is able to sense stimuli, store data, process signals, and actuate muscles. The soft turtle can switch swimming gaits after sensing twisting stimuli.
