# Supplementary material for: Reprogrammable, intelligent soft origami LEGO coupling actuation, computation, and sensing
Source: Innovation (Camb). 2023 Nov 29;5(1):100549. doi: 10.1016/j.xinn.2023.100549 (PMC10772819; doi:10.1016/j.xinn.2023.100549)
Supplement: Document S2. Article plus supplemental information [file mmc13.pdf]

ARTICLE

Reprogrammable, intelligent soft origami LEGO coupling actuation, computation, and sensing

Zhongdong Jiao,[1] Zhenhan Hu,[1] Yuhao Shi,[1] Kaichen Xu,[1] Fangye Lin,[1] Pingan Zhu,[1] Wei Tang,[1] Yiding Zhong,[1] Huayong Yang,[1] and Jun Zou[1,*]

*Correspondence: junzou@zju.edu.cn

Received: August 11, 2023; Accepted: November 28, 2023; Published data: November 29, 2023; https://doi.org/10.1016/j.xinn.2023.100549

© 2023 The Author(s). This is an open access article under the CC BY license (http://creativecommons.org/licenses/by/4.0/).

GRAPHICAL ABSTRACT

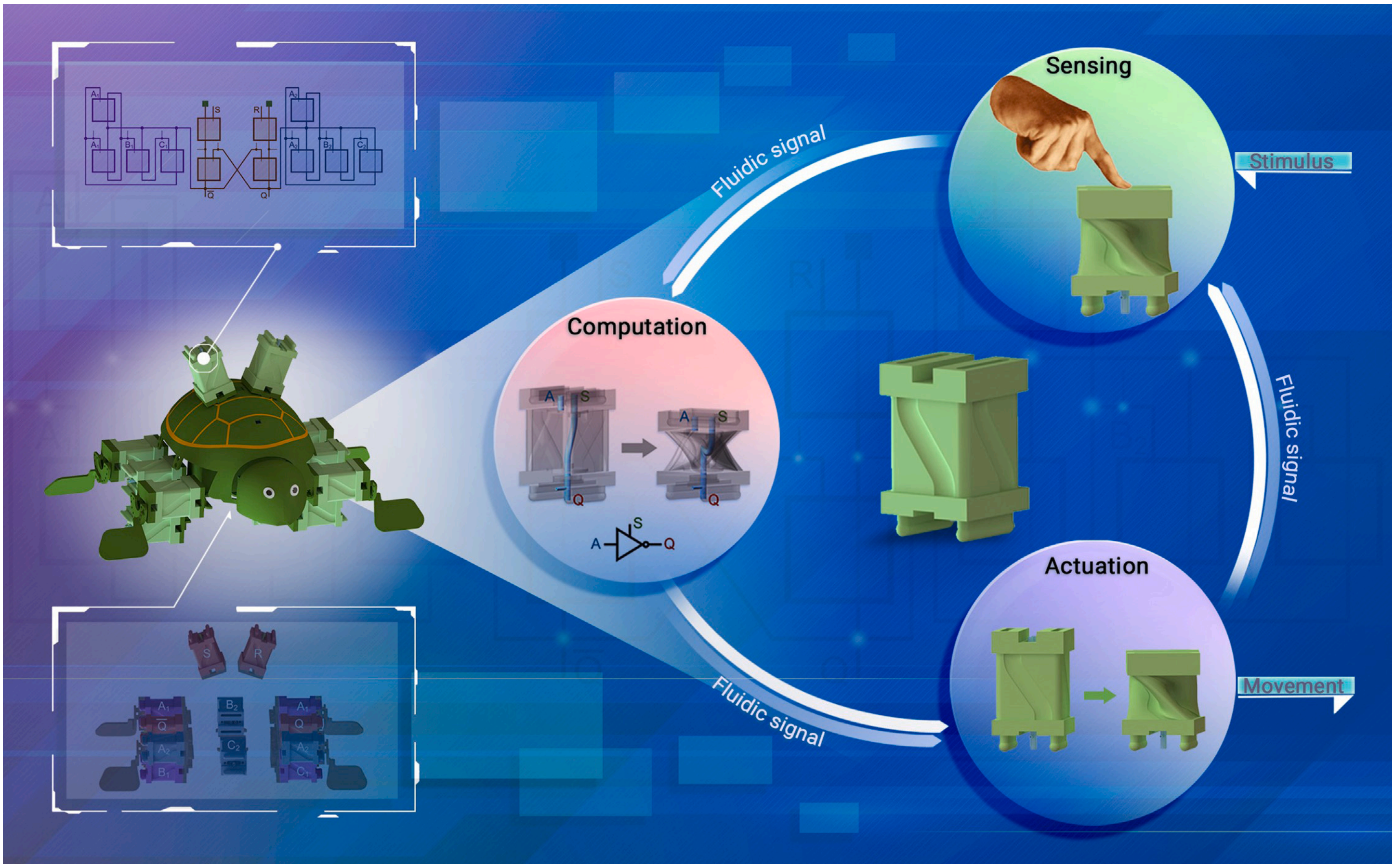

PUBLIC SUMMARY

- Soft origami LEGO coupling actuation, computation, and sensing.
- Reprogrammable soft robot morphologies and logic circuits can be readily created.
- Soft robots can sense stimuli, store data, process information, and actuate muscles.
- The soft origami possesses function multiplexing and signal compatibility.

Reprogrammable, intelligent soft origami LEGO coupling actuation, computation, and sensing

Zhongdong Jiao,[1] Zhenhan Hu,[1] Yuhao Shi,[1] Kaichen Xu,[1] Fangye Lin,[1] Pingan Zhu,[1] Wei Tang,[1] Yiding Zhong,[1] Huayong Yang,[1] and Jun Zou[1,*]

[1]State Key Laboratory of Fluid Power and Mechatronic Systems, Zhejiang University, Hangzhou 310058, China

*Correspondence: junzou@zju.edu.cn

Received: August 11, 2023; Accepted: November 28, 2023; Published data: November 29, 2023; https://doi.org/10.1016/j.xinn.2023.100549

© 2023 The Author(s). This is an open access article under the CC BY license (http://creativecommons.org/licenses/by/4.0/).

Citation: Jiao Z., Hu Z., Shi Y., et al., (2024). Reprogrammable, intelligent soft origami LEGO coupling actuation, computation, and sensing. The Innovation **5(1)**, 100549.

Tightly integrating actuation, computation, and sensing in soft materials allows soft robots to respond autonomously to their environments. However, fusing these capabilities within a single soft module in an efficient, programmable, and compatible way is still a significant challenge. Here, we introduce a strategy for integrating actuation, computation, and sensing capabilities in soft origami. Unified and plug-and-play soft origami modules can be reconfigured into diverse morphologies with specific functions or reprogrammed into a variety of soft logic circuits, similar to LEGO bricks. We built an untethered autonomous soft turtle that is able to sense stimuli, store data, process information, and perform swimming movements. The function multiplexing and signal compatibility of the origami minimize the number of soft devices, thereby reducing the complexity and redundancy of soft robots. Moreover, this origami also exhibits strong damage resistance and high durability. We envision that this work will offer an effective way to readily create on-demand soft robots that can operate in unknown environments.

INTRODUCTION

The seamless integration of actuation, computation, and sensing as exhibited by biological systems has long served as a source of inspiration for systems engineering. Imparting such capabilities to a single soft module enables various soft robots to readily be reconfigured to accommodate unknown situations. However, current advances have focused largely on individual components rather than multifunctionality integration. For example, a variety of novel soft actuators have been reported, ranging from fluidic actuators,[1–4] dielectric elastomers,[5,6] liquid crystal elastomers,[7] hydrogels,[8] and magnetic actuators,[9] to shape memory polymers.[10] In the field of soft controllers, soft components with embedded computation capability possess the ability to convert one constant input signal to multiple oscillatory output signals.[11–16] Advances in flexible sensors have expanded sensing capabilities to the detection of strain,[17] pressure,[18] temperature,[19] sweat,[20] and odor[21] using soft materials. With the current development in these fields, researchers such as Drotman et al.[11] have successfully assembled independent soft actuators, oscillators, and sensors into autonomous soft robots. Despite significant progress, the actuation, computation, and sensing capabilities of soft robots still depend on different components, which results in bulky systems, intricate fabrication processes, and poor reconfigurability.

An effective approach to solving these challenges is to implant computation and sensing capabilities into soft actuators. A variety of promising strategies, such as kirigami,[22,23] origami,[24,25] nonlinear actuators,[26–28] and viscous flow[29,30] have been harnessed to encode physical intelligence into soft materials and to program the actuation sequences of soft robots. Nonetheless, the intelligence and morphing enabled by these strategies are usually preprogrammed and can hardly be altered during in-life service, limiting the application of these robots in unstructured environments. In addition, soft sensors can be attached to an actuator surface or embedded into the actuator body.[31–35] These sensors detect external stimuli by measuring variations in resistance,[31,32] capacitance,[33] light power,[34] and magnetic fields.[35] However, the sensing signals generated are typically incompatible with the control signals of the aforementioned soft control devices. As a result, electronic conversion circuits are needed to bridge the difference between signals. Therefore, coupling actuation, computation, and sensing in an efficient, programmable, and compatible way has been an endless pursuit of researchers.

Biological systems have evolved to satisfy multiple needs with a single composite, for example, protein, which is the physical basis of organisms, is a typical example of multifunctionality. Even the same proteins are capable of exhibiting different functions, such as maintaining cell shape, catalyzing biochemical reactions, and ferrying nutrients across membranes. Inspired by these lessons, we present a reprogrammable intelligent soft origami (ReISO) that is endowed with integrated actuation, computation, and sensing. The origami is reconfigurable in morphology and reprogrammable in intelligence through a combination of multifunctionality and plug-and-play design, similar to LEGO bricks. Various function architectures, ranging from twisting, contraction, and bending to radial movement configurations, can be rapidly assembled with unified origami modules. Similarly, fundamental combinatorial and sequential logic circuits and complex functional circuits were also constructed. In addition, the strong damage resistance, high durability, low cost, and easy fabrication of the soft origami allow it to be easily mass-produced. Finally, we fabricated an untethered autonomous soft turtle that was capable of responding to environmental stimuli with only a soft control circuit. Soft actuators and sensors were fused into this circuit without introducing additional soft devices, which was realized via the signal compatibility and function multiplexing strategy of the origami.

RESULTS

Working principle of ReISO

Soft origami. The ReISO design is based on the Kresling origami,[25,36–38] which is a triangulated hollow cylinder. As illustrated in Figures 1A and 1B, the Kresling origami comprises two square panels for its upper and lower surfaces and eight triangular panels for its sides. When subjected to clockwise (CW) torque, this origami folds along its four inclined creases (the red dotted lines in Figures 1A and 1B), yielding a twist-contraction movement. Similarly, the ReISO possesses four slanted grooves, which function in the same way as the creases of the Kresling origami and split the square side into two triangular panels. The whole structure is a cubic airtight chamber, with one control tube inserted into it and one intelligent tube passing through it (Figures 1C and 1I). The plug-and-play male and female connectors are located on the upper and lower surfaces of the ReISOs to facilitate their assembly/disassembly, as shown in Figure S3.

Actuation capability. As shown in Figures 1C and 1D, when vacuum pressure is applied to the chamber via the control tube, the two triangular panels on the same side fold along the slanted groove. The simultaneous folding behavior on all four sides triggers a compound deformation that couples twisting and contraction. The ReISO is able to twist CW or anticlockwise (ACW), and its twisting direction is determined by the inclined direction of the slanted grooves, resembling the Kresling origami (Figure S4; Video S1). The twisting angle and contraction stroke increase with increased vacuum power, demonstrating the deformation controllability of the ReISOs (Figure S5). Furthermore, the soft origami has a fast response time of approximately 0.20 s (CW twisting) and 0.18 s (ACW twisting) to reach its deformed state (90% of the maximum twisting angle), as depicted in Figure S6.

Computation capability. It should be noted that this soft origami possesses computation capability, which is realized through the intelligent tube and capillary tube (Figures 1I and 1J). A ReISO consists of three logic ports: input port A, source port S, and output port Q. S and Q are the two ends of the intelligent tube. The capillary tube connects the output port Q with the atmosphere. In this work, we define the atmospheric pressure and vacuum pressure as the fluidic signals 0 and 1, respectively. When the origami is in its original state (the input port A is connected to the atmosphere), the intelligent tube in the chamber is straight and air flows freely through it (Figure 1I; Video S2). The source port S and output port Q are in the same pressure state (vacuum pressure). When the origami is actuated (the input port A is connected to vacuum pressure), the intelligent tube is kinked and becomes V-shaped, and the airflow is blocked

www.the-innovation.org

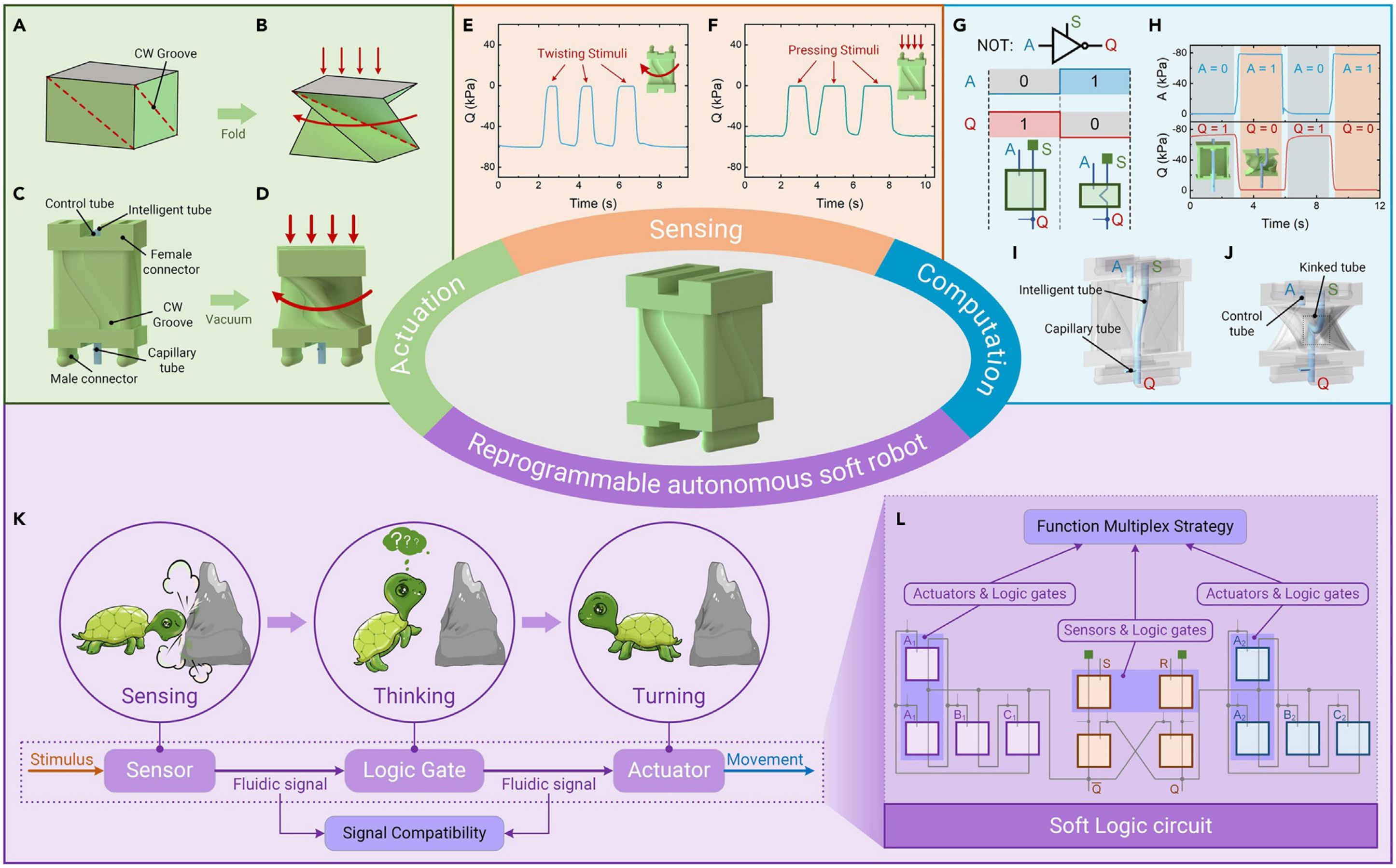

Figure 1. Reprogrammable intelligent soft origami (ReISO) with embedded physical intelligence (A and B) The clockwise Kresling origami is in unfolded (A) and folded (B) states. The red dashed lines represent the creases. The red arrows indicate the folding direction of the Kresling origami. (C and D) Schematic illustration of the clockwise ReISO in unfolded (C) and folded (D) states. The grooves in the sides denote the creases of the soft origami. (E and F) The pressure response of the ReISO when twisting stimuli (E) and pressing stimuli (F) are applied to it. (G) The ReISO is equivalent to a NOT logic gate. The bottom figure is the truth table of the soft NOT logic gate. A, S, and Q are the input port, source port, and output port, respectively. The green block represents the vacuum pressure. (H) The pressure response of the ReISO. The S port is connected to a constant vacuum pressure of −80 kPa. The atmospheric pressure is defined as fluidic signal 0, and the vacuum pressure is defined as fluidic signal 1. (I and J) The intelligent tube is in straight (I) and kinked (J) states. (K) An autonomous soft turtle built solely with ReISOs is able to sense, think, and move. The signal transmission between soft sensors, logic circuits, and actuators is enabled through fluidic signals. (L) The soft control system of the turtle. The modules in the pinkish-purple boxes are multiplexed as 2 functional components.

due to the axial force exerted on the intelligent tube (Figure 1J). The pressure state of the output port Q is converted from vacuum pressure to atmospheric pressure via the capillary tube. In this way, the soft origami converts the fluidic signal 1 to 0 or converts 0 to 1, which is the same function as a NOT logic gate (Figures 1G and 1H). The blocking behavior of ReISO also works with pressurized air, as shown in Figure S7. In this case, both the vacuum pressure and positive pressure are represented as 1.

Sensing capability. In addition to actuation and computation capabilities, soft origami exhibits a sensing capability, which is realized with the intelligent tube and capillary tube. Origami deformations can be obtained by measuring the pressure changes at port Q. As demonstrated in Figures 1E and 1F; Video S3, when the origami was twisted or pressed, the pressure at port Q increased owing to the kink of the intelligent tube. After releasing the mechanical stimuli, the pressure at port Q decreased.

As illustrated in Figures S20A and S20B, the pressure at port Q increases with an increase in the twisting angle α and compression L_c, and a nonlinear relationship between them can be observed. The ReISOs have a measuring range of 20°–65° for twisting angle and 1–10 mm for compression, respectively. In addition, they exhibit low hysteresis between the deformation and release processes.

A continuous fatigue test of twisting and compressing the ReISOs for 3,000 cycles was carried out (Figures S20C and S20D). The ReISOs exhibited consistent sensing capability, and no failures or permanent changes were observed during the testing, which suggests that the ReISOs had excellent durability and repeatability.

The deformation of the origami is measured as fluidic pressure signals, which are compatible with the actuation signals of fluidic actuators and the control signals of fluidic circuits (Figure 1K). Therefore, it is possible to fuse actuation, computation, and sensing functions within a soft intelligent system that is composed solely of ReISOs (Figure 1K). Furthermore, the function multiplexing strategy, in which a single ReISO can act as two soft components in one soft machine (Figure 1L), substantially reduces the redundancy of soft control systems, as described in detail in the following sections.

Logic characterization of the ReISOs

The computation capability of the origami is enabled via an intelligent tube that passes through the origami; thus, the geometric parameters of the tubes must be chosen carefully. The intelligent tubes have to satisfy two requirements: (1) they cannot be buckled under the vacuum state and (2) they should effectively block the airflow when compressed. To accommodate soft actuators with varying degrees of deformation, it is desirable to minimize the compression stroke (kinking threshold ΔH_{kink}) required to block airflow. This is because a smaller ΔH_{kink} endows the ReISOs with more reliable computation capability. Then, the kinking properties of elastomer tubes with different dimensions were investigated. As depicted in Figure 2A, thinner tubes (internal diameter × external diameter = 1.5 × 2.0 mm) collapsed when subjected to vacuum pressure (Figure S23A). Tubes with a size of 1.0 × 2.0 mm were difficult to be kinked due to their smaller internal and external diameters (Figure S23B). The remaining four types of tubes could be kinked with a compression stroke of less than 9 mm (Figure S23C). Notably, the tubes with a size of 2.0 × 3.0 mm exhibited the smallest kinking threshold ΔH_{kink} and were used as the intelligent tubes for the ReISOs.

For tubes with the same diameters, the kinking threshold ΔH_{kink} increases as the tube length increases (Figure 2B). When the ReISO is actuated, a torque is also exerted on the tube. We then studied the dependence of the kinking

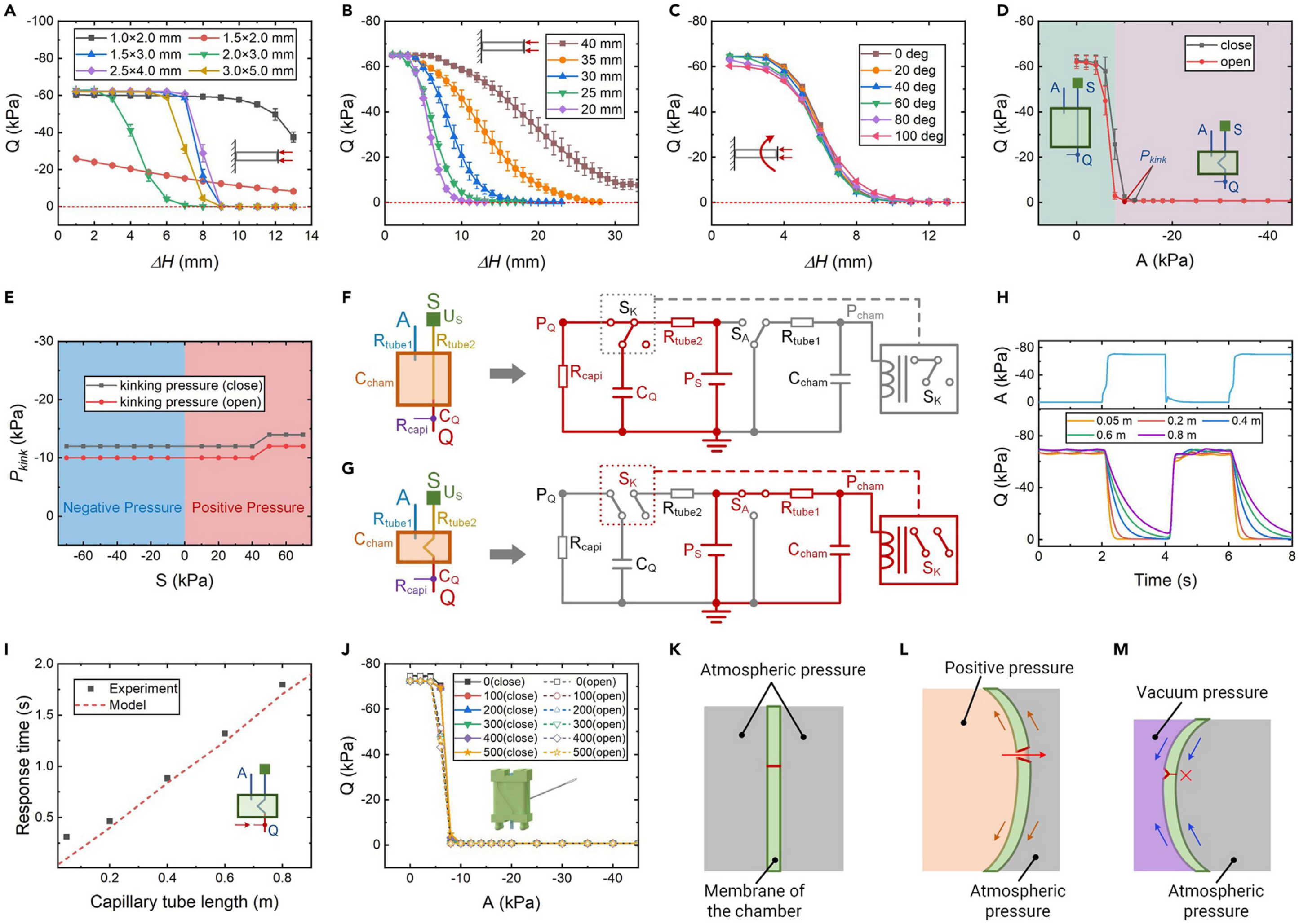

Figure 2. The logic performances of the ReISOs (A) The kinking characterization of elastomer tubes with different diameters. The numbers in the legend represent the internal and external diameters of the elastomer tubes. The unkinked length of these tubes is 20 mm. (B) The kinking characterization of elastomer tubes with different lengths. (C) The influence of the pretwisted angle of the elastomer tubes on the kinking characterization. (D) The relationship between the pressure at the output port Q and the pressure at the input port A. (E) The relationship between the kinking pressure and the pressure of the source port S. (F and G) The equivalent fluidic circuit of the ReISO in the unfolded (F) and folded (G) states. (H) The influence of the capillary tube length on the pressure response of the ReISOs. (I) The relationship between the capillary tube length and the response time of the ReISOs. (J) The pressure response of the ReISO after it is pricked with a needle. The numbers in the legend represent the number of needle pricks. (K–M) The damage-resistance principle of the ReISOs. The chamber of the ReISO is connected to the atmospheric pressure (K), positive pressure (L), and vacuum pressure (M), respectively. The red line represents the hole pricked by a needle. The orange and blue lines represent tension forces and compression forces, respectively. The error bars in this figure are calculated based on three tests.

threshold on the twisting angle of the soft origami by twisting a tube with a length of 20 mm from 0° to 100°. Interestingly, the kinking threshold shows little variation for different twisting angles (Figure 2C), suggesting that the blocking behavior is mainly attributed to the contraction of the ReISO.

We define kinking pressure P_{kink} as the pressure required to completely block the airflow in the intelligent tube. As shown in Figure 2D, the kinking pressure for closing the airflow is slightly lower than that for opening it. This hysteresis can be attributed to the snapping behavior that occurs during the compression of the tubes.

Furthermore, we investigated the influence of the pressure at the source port S on the kinking pressure. We observed that the pressure at port S had a slight influence on the kinking pressure, even when positive pressures were applied (Figure 2E). Higher positive pressures (≥45 kPa) resulted in greater forces applied to the internal surface of the intelligent tube; therefore, a larger compression force (lower vacuum pressure at port Q) is required to kink the tube.

Subsequently, we developed an analytical model to characterize the logic performances of the ReISOs. As shown in Figures 2F and 2G, the ReISO can be equivalent to a fluidic circuit, in which the fluidic pressure (Pa), mass flow rate (kg/s), fluidic resistance (Pa · s/kg), and fluidic capacity (kg/Pa) are analogous to the voltage (V), current (A), resistance (Ω), and capacity (F) of electrical circuits, respectively. Then, the logic response time of the ReISO can be expressed as Equation 1 (the detailed derivation is described in the supplemental information):

$$t_{1\rightarrow 0} = -R_{capi}C_Q \ln\frac{P_{Q1}}{P_{Q0}} + t_{kink} \quad \text{(Equation 1)}$$

where R_{capi} is the fluidic resistance of the capillary tube, C_Q is the fluidic capacity of the airtight channel that connects with port Q when the ReISO is in the folded state, and P_{Q0} is the pressure of port Q in the unfolded state. The state in which the pressure at port Q (P_Q) is higher than P_{Q1} is defined as the logic low state (here, the fluidic signals processed by the ReISO are vacuum pressures; thus, the magnitudes of P_Q and P_{Q1} are negative).

The response time of the ReISO was found to increase with the length of the capillary tube (Figure 2H). The model prediction and experimental data are compared in Figure 2I, where good agreement is illustrated, suggesting that the model could be used as an analytical tool to predict the performances of soft fluidic circuits and to guide the design of future soft robots.

Fluidic soft actuators have persistently been plagued by the threat of leakage, which hampers their functionality and reliability. Here, we carried out damage tests, pricking the origami with a needle repeatedly, to explore its resistance to leakage. The diameter of the needle for the pricking experiments was 0.8 mm. The ReISOs were pricked at different locations on their four side surfaces. As depicted in Figure 2J and Video S4, pricking the soft origami 500 times has a negligible impact on the kinking pressure required to open and

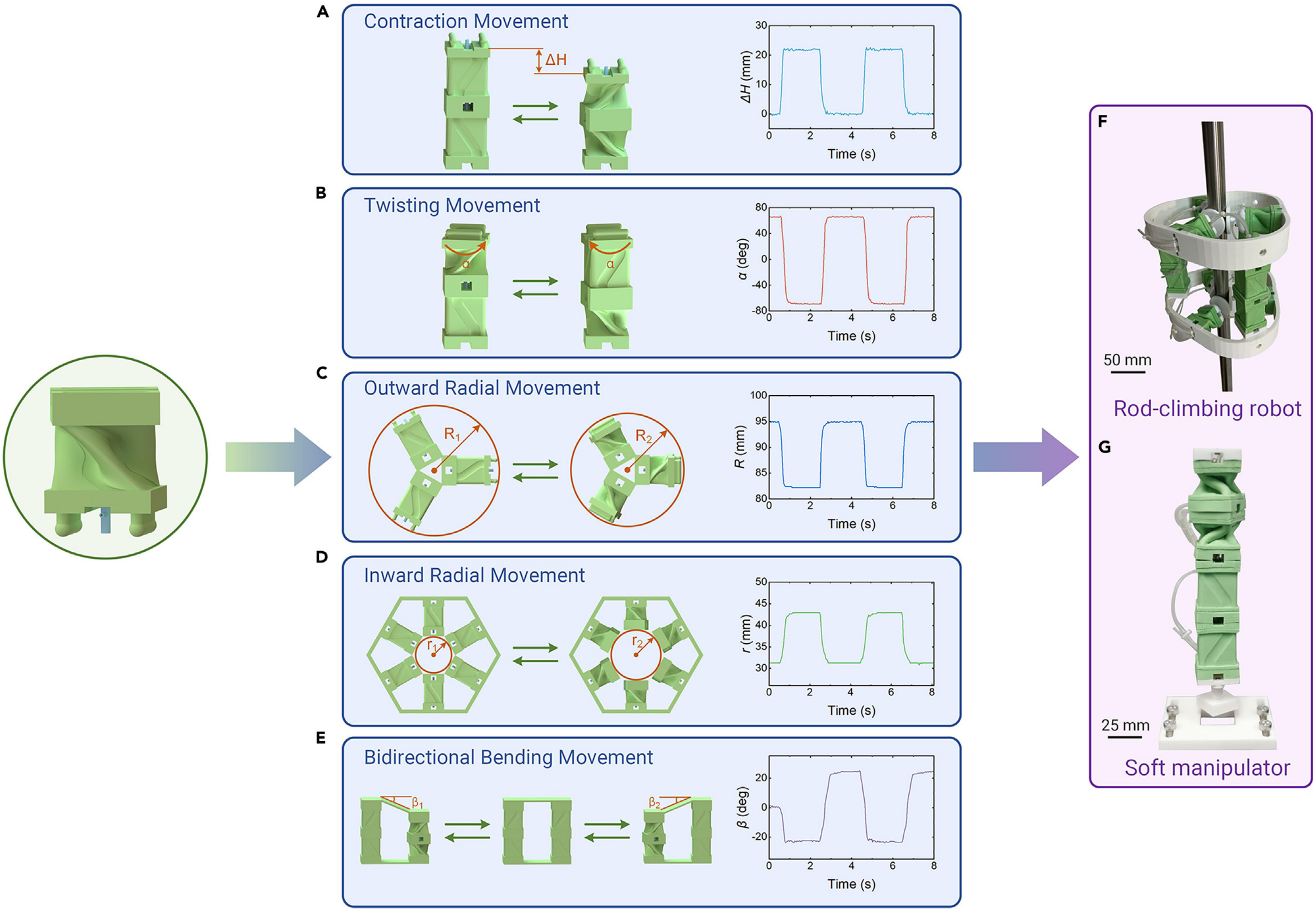

Figure 3. Reprogrammable morphologies of the ReISOs (A) Two ReISOs in the same actuation state form a contraction combination. (B) Two ReISOs in the opposite actuation states form a twisting combination. (C) An outward radial movement is realized by fixing 3 ReISOs in the same actuation state at the center of a circle. (D) An inward radial movement is achieved by fixing 6 ReISOs to a polygon that encircles them and actuating them simultaneously. (E) A bidirectional bending combination is enabled by assembling 2 contraction combinations in the opposite actuation states. (F) A soft rod-climbing robot constructed with the ReISOs. (G) A soft manipulator constructed with the ReISOs.

close the airflow. This excellent leak resistance is attributed to the unique buckling behavior of the ReISOs. As shown in Figures 2K–2M, the positive pressure causes the size of the pinhole to increase owing to the tension forces in the membrane, whereas the vacuum pressure decreases the size of the pinhole due to the compression forces in the membrane. Consequently, our vacuum-powered ReISOs exhibit better leak resistance than do fluidic soft actuators that are driven by pressurized air.

Reconfigurable morphologies

A single ReISO is able to exhibit a compound deformation that couples twisting and contraction movements. This unique characteristic of the ReISO, combined with its plug-and-play connectors, allows for the facile reconfiguration of a vast array of morphologies with specific motion behaviors (Video S5). As illustrated in Figures 3A and 3B, two ReISOs with opposite twisting directions are connected in series, forming a morphology that can perform pure contraction or pure twisting movements. The simultaneous actuation of the two ReISOs counteracts their twisting deformations, resulting in a pure contraction movement. Conversely, actuating one ReISO and releasing the other one counteracts their contraction deformations, which yields a pure twisting movement. When several (three or more, in this case, we use three) ReISOs are evenly distributed along the circumferential direction of a circle and fixed at the center of the circle, their simultaneous actuation produces an outward radial movement (Figure 3C). By fixing these ReISOs (in this case, six modules are used) to a polygon that encircles them, an inward radial movement can be generated, as shown in Figure 3D. Assembling these basic combinations into more complex ones can unlock movements with greater diversity. For example, two contraction combinations can exhibit a bidirectional bending movement, as demonstrated in Figure 3E.

By using these ReISO-based movement combinations, we successfully fabricated two soft robots that possessed different functions. The first one was a rod-climbing robot, which consisted of two inward radial combinations and a contraction combination, as illustrated in Figure 3F. The inward radial combination allowed the robot to grasp and loosen the rod, whereas the contraction combination offered forward/backward thrust for the robot. This rod-climbing robot was able to climb along a smooth rod at a speed of 2.67 mm/s (Video S6). The second robotic prototype was a soft manipulator capable of placing objects with specific shapes into holes. As depicted in Figure 3G and Video S6, this device was composed of a contraction combination (the upper two modules) and a twisting combination (the lower two modules). The twisting combination adjusted the orientation of the objects so that the objects matched the shape of the holes, and the contraction combination subsequently dropped the objects into the holes. The twisting angle of the ReISOs could be tuned by regulating the actuation pressure, as demonstrated in Figure S5.

Reprogrammable intelligence

Reprogrammable soft combinatorial logic circuits. A single ReISO serves as a NOT gate, which is a functionally complete binary logic gate and is able to construct all the fundamental 1-bit (NOT and Buffer) and 2-bit (NAND, NOR, AND, OR, XOR, and XNOR) logic gates via the assemblage of multiple modules (Video S7). For instance, the Buffer gate is an inverted NOT gate and can be created from a two-module combination (Figure 4A), in which the output port of the first ReISO is connected to the input port of the second ReISO ($A = \overline{\overline{A}}$).

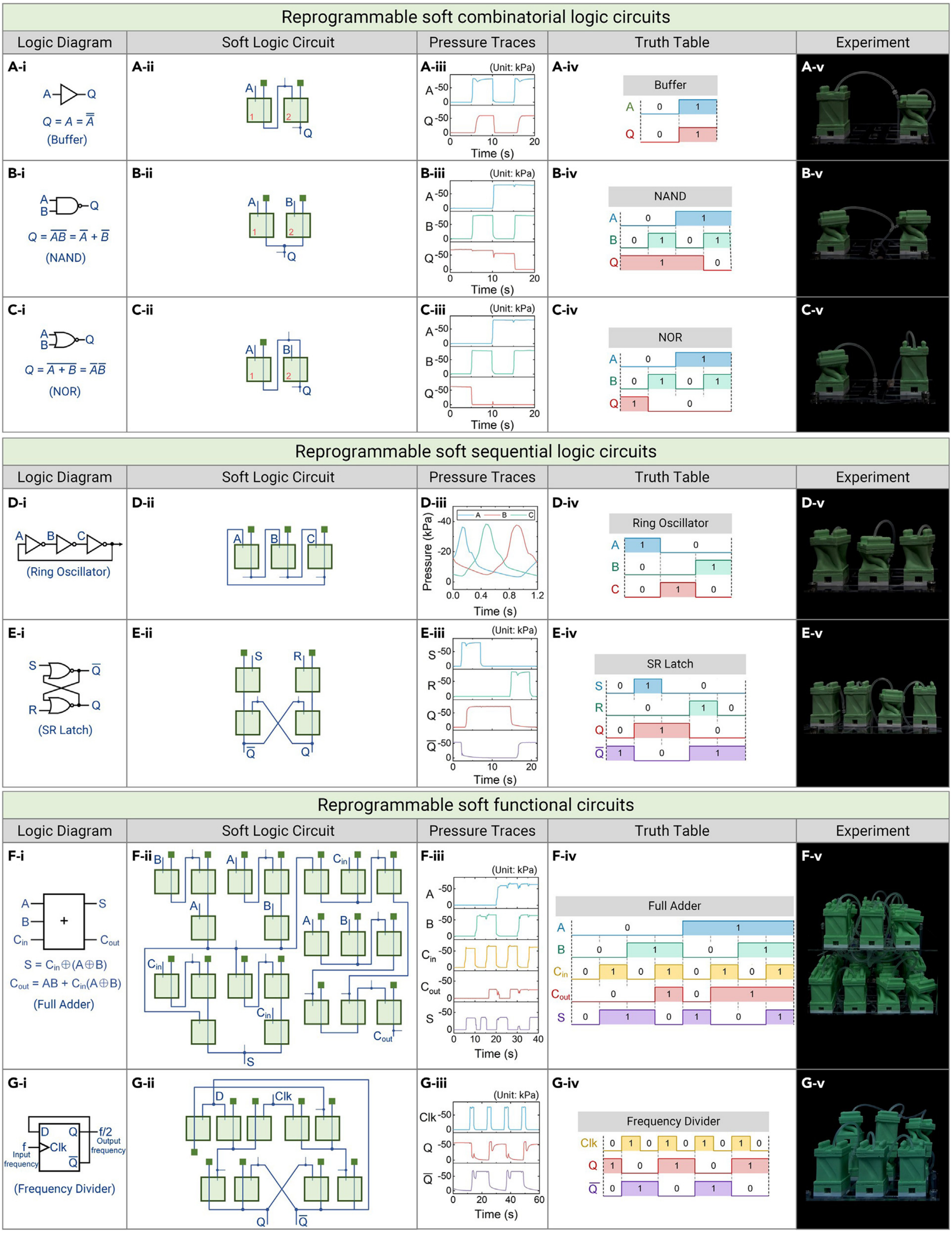

Figure 4. Reprogrammable soft logic circuits of ReISOs (A–C) The soft combinatorial logic circuits. (A-i) The logic symbol and Boolean expression of the Buffer gate. (A-ii) The schematic of the soft Buffer gate circuit. (A-iii) The pressure traces of the soft Buffer gate. (A-iv) The truth table of the Buffer gate. (A-v) The experimental image of the soft Buffer gate. (B) The NAND gate. (C) The NOR gate. (D and E) The soft sequential logic circuits. (D) The soft ring oscillator circuit. (E) The soft SR latch circuit. (F and G) The soft functional circuits constructed with the above fundamental logic circuits. (F) The soft full adder circuit. (G) The soft frequency divider circuit. The green block represents the vacuum pressure.

The NAND and NOR gates can be expressed using two NOT gates, according to De Morgan's theorems. As shown in Figure 4B, the NAND gate is constructed by connecting the output ports of two ReISOs ($\overline{AB} = \overline{A} + \overline{B}$), whereas the NOR gate is built by connecting the output port of the first ReISO to the source port of the second ReISO ($\overline{A+B} = \overline{A}\,\overline{B}$; Figure 4C). The AND gate and OR gate are the inversions of the NAND gate and NOR gate, respectively. Thus, they can be obtained by combining the NAND gate and NOR gate with a NOT gate ($AB = \overline{\overline{AB}}, A+B = \overline{\overline{A+B}}$), respectively (Figures S14A and S14B). Similarly, XOR and XNOR gates can be built with six NOT gates ($A \oplus B = A\overline{B} + \overline{A}B, A \odot B = \overline{A}\,\overline{B} + AB$), as shown in Figures S14C and S14D.

Reprogrammable sequential logic circuits. In addition to combinatorial logic circuits, sequential logic circuits can also be realized with ReISOs. Unlike combinatorial logic circuits, which rely only on present inputs to generate outputs, sequential logic circuits depend on both present and past inputs to generate outputs.

A ring oscillator is a sequential logic circuit that is able to "process" input signals. As shown in Figures 4D–i, it is created by connecting an odd number of NOT gates as a loop, with the output of the last NOT gate fed back into the first one. Then, the last output is the logical NOT of the first input due to the odd number of NOT gates. In Figures 4D–ii, we used three ReISOs to build a soft ring oscillator. When the source ports of the three ReISOs were connected to a constant vacuum pressure, the loop connection enabled the three ReISOs to oscillate spontaneously and sequentially between their unfolded and folded states (Video S8). The working principle of the soft ring oscillator is described in the supplemental information. As a result, the constant pressure was converted into multiple (odd numbered) oscillatory pressures, verifying the signal processing capability of ReISOs.

The SR latch is a logic circuit with a 1-bit memory and is able to "store" previous input values. As shown in Figures 4E–i, this circuit accepts two inputs (S and R) and provides two complementary outputs (Q and $\overline{Q}$). The input S will SET the device (meaning the output Q = 1), whereas the input R will RESET the device (meaning the output Q = 0). In Figures 4E–ii, we used two cross-coupled NOR gates to build an SR latch, in which the output of one NOR gate is fed back to the input of the other one and vice versa. As shown in Figures 4E-iii and 4E-iv and Video S8, setting the input S to 1 switched the output Q to 1, which remained 1 even after the input S was returned to 0, functioning as a memory device. Likewise, setting the input R to 1 switched the output Q to 0, which maintained 0 after the input R was returned to 0. Therefore, this soft circuit is able to remember previous input signals and exhibit memory functionality.

Furthermore, we also constructed a soft JK flip-flop circuit and a soft D flip-flop circuit to exhibit the capability of the ReISOs in reprogramming sequential logic circuits. These circuits are described in the supplemental information and Figure S16.

Reprogrammable soft functional circuits. With these basic logic circuits, any higher-level functional logic circuits could, in principle, be programmed by harnessing the foundations of canonical Boolean functions and their algebraic combinations. To verify this capability, the implementation of a soft full adder and a soft frequency divider is taken as an example (Figures 4F and 4G; Video S9).

As illustrated in Figures 4F-i, a full adder is an arithmetic circuit that contains three inputs, namely, A, B, and C_{in} (carry input), and two outputs, C_{out} (carry output) and S. The two outputs can be expressed with the fundamental logic gates mentioned above: $S = C_{in} \oplus (A \oplus B)$, $C_{out} = AB + C_{in}(A \oplus B)$. (The Boolean operation symbols are depicted in Figures 4A–4C and S14) Next, the soft full adder was fabricated by assembling 21 ReISOs and connecting them according to the logic circuit shown in Figure 4F-ii. All eight addition computations possible for the full adder were experimentally validated (Figures 4F-iii and 4F-iv; Video S9), demonstrating the number operation functionality of ReISOs. The soft frequency is described in the supplemental information.

Autonomous and reconfigurable soft robots

Reconfigurable soft robots with built-in intelligence. As shown in Figures 5A and 5B, we built a soft robotic turtle that is made up of five ReISOs, a miniature vacuum pump, and a lithium battery. The ReISOs with the same label are connected; thus, they are always in the same actuation state and same logic state. In this robot, the two ReISOs connected with the front legs of the turtle are labeled C, the two ReISOs connected with the hind legs are labeled B, and the ReISO in the center of the robot body is labeled A. The five ReISOs form a soft ring oscillator circuit composed of three NOT logic gates, which acts as the soft controller of the turtle (Figure 5C). This controller converts the constant pressure generated by the vacuum pump into three oscillatory pressures, causing the five ReISOs to fold and unfold periodically. Consequently, the four ReISOs connected with the legs of the turtle also serve as actuators: they generate the rhythmic swinging of the legs. The swinging speed during the folding process is faster than that during the unfolding process (Figure 5D). Therefore, the thrust generated during the folding process is larger than that during the unfolding process (the operation principle is described in the supplemental information), driving the turtle to rhythmically move forward. The soft turtle can be reprogrammed to swim forward, backward, CW, and ACW by reconfiguring modules B and C (Figures 5F–5I; Video S10, the orange lines in Figures 5F–5I represent the twisting direction of the modules). The swimming and rotating speeds that the soft turtle can reach are 24.65 mm/s (5.4 body length/min) and 7.77°/s, respectively.

Autonomous soft robots. To adapt to the dynamic and unstructured environment, soft robots are also required to sense external stimuli and perform multiple movement gaits. Then, the sensing capability of the ReISO is introduced into the soft turtle (Figure 6A). It should be noted that, when acting as a soft sensor, the ReISO is able to output fluidic signals, which are compatible with the control system described above (Figure 1K). We constructed a more advanced soft turtle that was capable of sensing stimuli, storing data, processing signals, and actuating muscles using only ReISOs. As illustrated in Figures 6B and 6C, the turtle consists of an SR latch circuit and two ring oscillator circuits, with the source ports of the two ring oscillators connected to the output ports of the SR latch ($\overline{Q}$ and Q). The SR latch circuit is responsible for sensing external stimuli and storing data, whereas the two ring oscillator circuits convert the constant pressure from the output ports of the SR latch circuit to oscillatory pressures. Meanwhile, ring oscillator 1 is connected to the hind legs to enable backward swimming, and ring oscillator 2 is connected to the front legs to enable forward swimming. The SR latch circuit ensures that only one ring oscillator is activated at a time, resulting in the swinging motion of a single pair of legs in the water. It is interesting to note that the number of soft devices required to actuate this soft turtle was minimized because the soft sensors and actuators were integrated into the SR latch circuit and ring oscillator circuits, respectively, which is the function multiplexing strategy of ReISOs (the green area in Figure 6B).

As depicted in Figures 6D, 6E, and S17A–S17C and Video S11, when the module S was subjected to a CW torque (>0.087 N · m, illustrated in Figure S22), the turtle detected this torque stimulus. Then, the output Q of the SR latch circuit (the orange circuit in Figure 6B) was set to 1 (in this state, the module $\overline{Q}$ is in the folded state), providing vacuum power for ring oscillator 2. Releasing the CW torque did not change the state of Q because the previous state was stored in the SR latch circuit. The logic high state of Q allowed the two front legs to be actuated by the module A_2 of ring oscillator 2 (the blue circuit in Figure 6B), and the turtle began to swim forward. Likewise, when the ACW torque was applied to the module R, the output $\overline{Q}$ became a logic high state and the turtle began to swim backward by the ring oscillator 1 (Figures 6E, 6F, and S17D–S17F). The stimuli sensing and data storing capabilities enabled the soft turtle to repeatedly switch between movement gaits. The stimuli type and data capacity could be increased further by reconfiguring the soft control system, thereby exhibiting more abundant movement gaits.

DISCUSSION AND CONCLUSION

In summary, a ReISO capable of actuating, computing, and sensing was proposed and characterized. This multifunctionality, combined with the modularity, endowed the ReISO with reconfigurable morphology and reprogrammable intelligence. A variety of motions including but not limited to contraction, twisting, bending, and radial motions was readily obtained and reconfigured. Using the embedded intelligence, we constructed a series of fundamental combinatorial logic circuits (NOT, Buffer, AND, OR, NAND, NOR, XOR, and XNOR) and sequential logic circuits (ring oscillator, SR latch, JK flip-flop, and D flip-flop). Next, a soft full adder and a soft frequency divider were presented to demonstrate the potential of the ReISO in building complex functional circuits. Moreover, this soft origami also featured high durability, low cost, easy fabrication, and strong damage resistance. The function multiplexing and signal compatibility of the ReISOs made it possible to build efficient and intelligent soft machines. As a proof of concept, an untethered autonomous soft turtle that was able to sense stimuli, store data, process information, and perform swimming movements was developed. We believe that the concept presented in this work will inspire and enable more intelligent soft robots with specialized functions and improved environmental adaptability.

Autonomous soft robots with built-in intelligence have attracted tremendous research interest recently. Compared with previous work, our ReISOs have several distinctive advantages, as listed in Table 1.

(1) Previous work[11–15,39–43] focuses on developing various soft control components for soft robots. These components are then combined

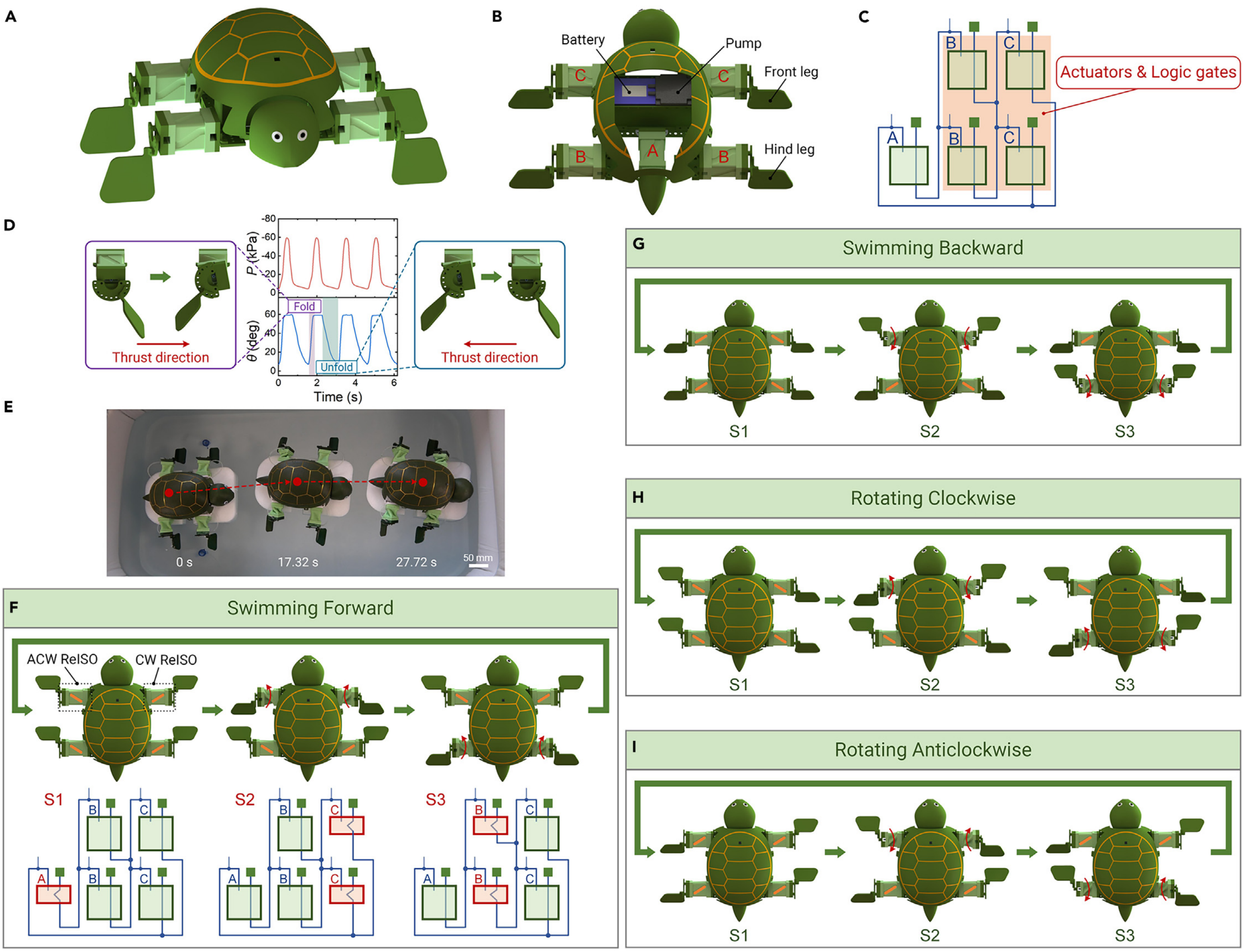

Figure 5. Reconfigurable soft turtle with built-in intelligence (A) Schematic illustration of the soft turtle. (B) The actuation system of the soft turtle. (C) The soft control circuit of the robotic turtle is a ring oscillator with 3 NOT gates. The ReISOs in the red area function as actuators and logic gates simultaneously. (D) The locomotion principle of the soft turtle. The curves are the pressure and twisting angle variations of the soft leg. (E) A sequence of images of the untethered soft turtle swimming forward in a tank. (F–I) The schematic actuation system of the soft turtle when it swims forward (F), backward (G), CW (H), and ACW (I). These motion modes can be readily achieved by reconfiguring ReISOs.

with additional soft actuators that are specifically designed to build soft robots. Unlike these strategies, we implanted physical intelligence into modular soft actuators to render them new functionalities that are rarely achieved in traditional soft actuators, including computation and sensing. The two additional functionalities are effectively enabled by adding an intelligent tube and a capillary tube, which satisfies two requirements with a simple structure and has no influence on the deformation of the actuator. The multifunctionality enables soft robots to sense stimuli, store data, process information, and actuate muscles using only ReISOs. This capability is especially advantageous in reconfiguring on-demand soft machines to adapt to unpredictable environments. When the ReISOs act as actuators, logic gates, and sensors, their input/output signals are the fluidic type (Figure 1K), sidestepping the signal incompatibility that exists in current intelligent soft robots. The compatible communication and integrated multifunctionality eliminate the need for additional microcontrollers, valves, and sensors, thereby decreasing the overall complexity of the autonomous soft robots with embedded intelligence. Furthermore, this multifunctionality integration strategy can readily be generalized to other fluidic actuators, accelerating the development of smart actuators.

(2) Although the soft-legged quadruped robot designed by Drotman et al.[11] is also able to respond to sensor input and switch movement gaits, it requires an additional soft sensor and four additional soft legs with three pneumatic chambers, which are larger than the soft control system. This robot can only switch its movement gait once, because only one sensor (a bistable valve) is introduced into the control system. By comparison, our soft turtle can respond to sensor input and switch movement gaits without additional soft components via the function multiplexing strategy. This strategy permits a single ReISO to perform multiple functions at the same time. For example, the modules S and R of the turtle function as soft sensors and logic gates simultaneously. The modules A_1 and A_2 act as actuators and logic gates simultaneously (Figures 6B and 6C). Consequently, the soft turtle only requires a control system (an SR latch circuit and two ring oscillator circuits) to achieve the same function as the soft-legged quadruped robot.[11] In addition, our soft turtle is able to switch movement gaits repeatedly via the data storing capability enabled by the SR latch circuit.

(3) Soft robots with both reconfigurable architectures and reprogrammable intelligence can be realized using the ReISOs. These modules can be assembled into different morphologies, allowing their compound deformation to be decoupled into pure twisting and pure contraction, or to be combined into other complex deformations. This reconfigurability widens the range of the possible movements that ReISOs can exhibit, permitting them to be configured as various soft robots to adapt to dynamic environments. The reprogrammable

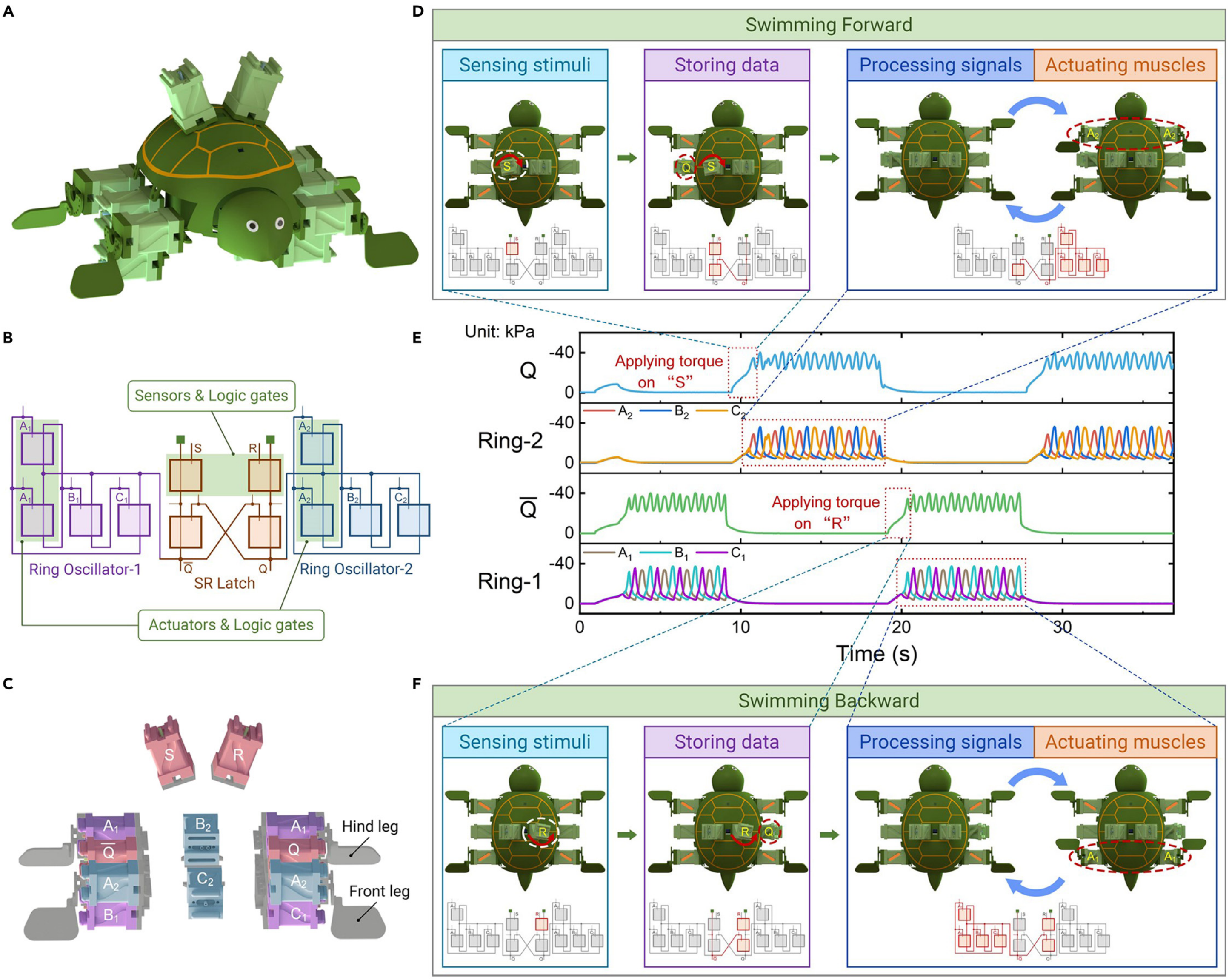

Figure 6. An untethered and autonomous soft turtle that is able to sense stimuli, store data, process signals, and perform swimming movements (A) Schematic illustration of the soft turtle. (B) The soft control system of the robotic turtle is composed of 2 ring oscillator circuits and an SR latch circuit. The ReISOs in the green area function as 2 components simultaneously. (C) The soft turtle was controlled and actuated with 12 ReISOs. The ReISOs with the same color belong to the same subcircuit. (D) The soft turtle switched to forward movement gaits after sensing a CW twisting stimulus. The SR latch circuit detected this stimulus and stored the current state in the circuit even if the stimulus was removed. The ring oscillators then converted the constant pressure from the output ports of the SR latch to oscillatory pressures, which were used to actuate the legs to swing in the water. The bottom figures are detailed information on the soft control system and are clearly depicted in Figure S17. (E) The pressure traces of the soft turtle. Q and $\overline{Q}$ represent the output pressure of the SR latch circuit. Ring-1 and Ring-2 denote the output pressures of ring oscillator 1 and ring oscillator 2. (F) The soft turtle switched to backward movement gaits after sensing an ACW twisting stimulus.

intelligence means that the ReISOs can be used to construct various soft combinatorial and sequential logic circuits. These circuits endow soft machines with the ability to switch between movement gaits according to external stimuli, execute number operations, and divide signal frequency. Similarly, other advanced functionalities, such as the soft counter, which stores the times a particular event has occurred, can also be achieved by reprogramming logic circuits. Furthermore, the plug-and-play connectors enable ReISOs to be readily assembled and disassembled, further enhancing their reconfigurability and reprogrammability.

(4) The ReISOs are standardized components, just like LEGO blocks. This modularity is especially advantageous in testing soft machines with new configurations, repairing/replacing damaged modules, and rapidly reconstructing soft machines in unstructured environments. In addition, the ReISOs can be fabricated via elastomer casting or liquid crystal display printing, which decreases their fabrication difficulty and cost. The multifunctionality, simple structure, easy fabrication, high durability, and low cost allow ReISOs to be produced in large quantities. Finally, the modular ReISOs have the potential to be used as commercial educational outfits for robotics learning.

(5) Current soft control devices[11–15,39–43] are typically actuated with positive pressure, which tends to cause air leakage and hinders their durability. By contrast, the ReISOs presented in this work are actuated with vacuum pressure and are resistant to pricking damage. This is because their inward collapse compresses the pinholes of the soft origami, thereby blocking the leakage of air (Figure 2M). The excellent damage resistance makes them especially suitable for applications in hazardous environments where pointed objects, such as nails or sharp stones, may pose a threat.

The ReISOs illustrated so far are centimeter-scale structures, limiting their applications in constrained spaces, such as the gastrointestinal tract, heart, and nasal cavity. However, recent developments in microscale fabrication and actuation[44] present an opportunity to downscale the intelligent soft actuators to microscale structures. As a soft sensor, the ReISO exhibits the ability to detect pressing or twisting stimuli. Nevertheless, the practical environment necessitates

Table 1. Capability comparison of our ReISOs with previous soft control devices

Capability	Soft valve[11,13-15,39,40]	Textile-based logic gate[41]	Tube-balloon logic gate[42,43]	Buckling-sheet ring oscillator[12]	This work
Actuation	–	–	–	✓	✓
Sensing	–	–	–	–	✓
Function multiplexing	–	–	–	✓	✓
Reconfigurable morphologies	–	–	–	–	✓
Plug-and-play	–	–	–	–	✓
Unified modules	–	–	–	✓	✓
Leak resistance	–	–	–	–	✓
Pressure type	Positive	Positive	Positive	Positive	**Positive Vacuum**
Low-cost, easy fabrication	–	✓	✓	✓	✓

the ReISO to be highly sensitive to obstacles. One potential way to improve sensing sensitivity is to use electrically driven soft actuation technologies, such as dielectric elastomer actuation,[5,6] electrohydrodynamic actuation,[45,46] and hydraulic electrostatic actuation,[3,47,48] to construct soft modules coupling sensing, computation, and actuation. By harnessing such technologies, the contact of two conductive membranes could trigger the reversal of motion directions. In this work, we used a commercial pump and battery to drive the soft turtle, which decreases the compliance of soft robots. Nevertheless, these hard components, such as the pneumatic battery powered by chemical reactions, could be replaced with soft power devices in our future work.[49]

MATERIALS AND METHODS

See the supplemental information for details.

DATA AND CODE AVAILABILITY

The data that support the findings of this study are available from the corresponding author upon reasonable request.

REFERENCES

1. Siéfert, E., Reyssat, E., Bico, J., et al. (2019). Bio-inspired pneumatic shape-morphing elastomers. Nat. Mater. ***18***, 24–28.
2. Jiao, Z., Zhang, C., Wang, W., Pan, M., Yang, H., and Zou, J. (2019). Advanced artificial muscle for flexible material-based reconfigurable soft robots. Adv. Sci. ***6***, 1901371.
3. Acome, E., Mitchell, S.K., Morrissey, T.G., Emmett, M.B., Benjamin, C., King, M., Radakovitz, M., and Keplinger, C. (2018). Hydraulically amplified self-healing electrostatic actuators with muscle-like performance. Science ***359***, 61–65.
4. Jiao, Z., Zhang, C., Ruan, J., Tang, W., Lin, Y., Zhu, P., Wang, J., Wang, W., Yang, H., and Zou, J. (2021). Re-foldable origami-inspired bidirectional twisting of artificial muscles reproduces biological motion. Cell Rep. Phys. Sci. ***2***, 100407.
5. Gu, G., Zou, J., Zhao, R., Zhao, X., and Zhu, X. (2018). Soft wall-climbing robots. Sci. Robot. ***3***, eaat2874.
6. Shi, Y., Askounis, E., Plamthottam, R., Libby, T., Peng, Z., Youssef, K., Pu, J., Pelrine, R., and Pei, Q. (2022). A processable, high-performance dielectric elastomer and multilayering process. Science ***377***, 228–232.
7. Liu, H., Tian, H., Li, X., Chen, X., Zhang, K., Shi, H., Wang, C., and Shao, J. (2022). Shape-programmable, deformation-locking, and self-sensing artificial muscle based on liquid crystal elastomer and low melting point alloy. Sci. Adv. ***8***, eabn5722.
8. Gao, G., Wang, Z., Xu, D., Wang, L., Xu, T., Zhang, H., Chen, J., and Fu, J. (2018). Snap-buckling motivated controllable jumping of thermo-responsive hydrogel bilayers. ACS Appl. Mater. Interfaces ***10***, 41724–41731.
9. Sun, M., Tian, C., Mao, L., Meng, X., Shen, X., Hao, B., Wang, X., Xie, H., and Zhang, L. (2022). Reconfigurable magnetic slime robot: deformation, adaptability, and multifunction. Adv. Funct. Mater. ***32***, 2112508.
10. Zhang, Q., Kuang, X., Weng, S., Yue, L., Roach, D.J., Fang, D., and Qi, H.J. (2021). Shape-memory balloon structures by pneumatic multi-material 4D printing. Adv. Funct. Mater. ***31***, 2010872.
11. Drotman, D., Jadhav, S., Sharp, D., Chan, C., and Tolley, M.T. (2021). Electronics-free pneumatic circuits for controlling soft-legged robots. Sci. Robot. ***6***, eaay2627.
12. Lee, W.K., Preston, D.J., Nemitz, M.P., Nagarkar, A., MacKeith, A.K., Gorissen, B., Vasios, N., Sanchez, V., Bertoldi, K., Mahadevan, L., and Whitesides, G.M. (2022). A buckling-sheet ring oscillator for electronics-free, multimodal locomotion. Sci. Robot. ***7***, eabg5812.
13. Preston, D.J., Jiang, H.J., Sanchez, V., Rothemund, P., Rawson, J., Nemitz, M.P., Lee, W.K., Suo, Z., Walsh, C.J., and Whitesides, G.M. (2019). A soft ring oscillator. Sci. Robot. ***4***, eaaw5496.
14. Hubbard, J.D., Acevedo, R., Edwards, K.M., Alsharhan, A.T., Wen, Z., Landry, J., Wang, K., Schaffer, S., and Sochol, R.D. (2021). Fully 3D-printed soft robots with integrated fluidic circuitry. Sci. Adv. ***7***, eabe5257.
15. Wehner, M., Truby, R.L., Fitzgerald, D.J., Mosadegh, B., Whitesides, G.M., Lewis, J.A., and Wood, R.J. (2016). An integrated design and fabrication strategy for entirely soft, autonomous robots. Nature ***536***, 451–455.
16. Jin, L., Forte, A.E., and Bertoldi, K. (2021). Mechanical valves for on-board flow control of inflatable robots. Adv. Sci. ***8***, 2101941.
17. Lyu, Q., Gong, S., Lees, J.G., Yin, J., Yap, L.W., Kong, A.M., Shi, Q., Fu, R., Zhu, Q., Dyer, A., Dyson, J.M., Lim, S.Y., and Cheng, W. (2022). A soft and ultrasensitive force sensing diaphragm for probing cardiac organoids instantaneously and wirelessly. Nat. Commun. ***13***, 7259.
18. Yu, P., Li, X., Li, H., Fan, Y., Cao, J., Wang, H., Guo, Z., Zhao, X., Wang, Z., and Zhu, G. (2021). All-fabric ultrathin capacitive sensor with high pressure sensitivity and broad detection range for electronic skin. ACS Appl. Mater. Interfaces ***13***, 24062–24069.
19. Park, J., Kim, M., Lee, Y., Lee, H.S., and Ko, H. (2015). Fingertip skin–inspired microstructured ferroelectric skins discriminate static/dynamic pressure and temperature stimuli. Sci. Adv. ***1***, e1500661.
20. Park, H.J., Jeong, J., Son, S.G., Kim, S.J., Lee, M., Kim, H.J., Jeong, J., Hwang, S.Y., Park, J., Eom, Y., and Choi, B.G. (2021). Fluid-dynamics-processed highly stretchable, conductive, and printable graphene inks for real-time monitoring sweat during stretching exercise. Adv. Funct. Mater. ***31***, 2011059.
21. Ozer, E., Kufel, J., Biggs, J., Rana, A., Rodriguez, F.J., Lee-Clark, T., Sou, A., Ramsdale, C., White, S., Garlapati, S.K., Valliappan, P., Rahmanudin, A., Komanduri, V., Saez, G.S., Gollu, S., Brown, G., Dudek, P., Persaud, K.C., Turner, M.L., Murray, S., Bates, S., Treloar, R., Newby, B., and Ford, J. (2023). Malodour classification with low-cost flexible electronics. Nat. Commun. ***14***, 777.
22. Rafsanjani, A., Jin, L., Deng, B., et al. (2019). Propagation of pop ups in kirigami shells. Proc. Natl. Acad. Sci. USA ***116***, 8200–8205.
23. Rafsanjani, A., Zhang, Y., Liu, B., Rubinstein, S.M., and Bertoldi, K. (2018). Kirigami skins make a simple soft actuator crawl. Sci. Robot. ***3***, eaar7555.
24. Bhovad, P., Kaufmann, J., and Li, S. (2019). Peristaltic locomotion without digital controllers: exploiting multi-stability in origami to coordinate robotic motion. Extreme Mech. Lett. ***32***, 100552.
25. Melancon, D., Forte, A.E., Kamp, L.M., Gorissen, B., and Bertoldi, K. (2022). Inflatable origami: multimodal deformation via multistability. Adv. Funct. Mater. ***32***, 2201891.
26. Gorissen, B., Milana, E., Baeyens, A., Broeders, E., Christiaens, J., Collin, K., Reynaerts, D., and De Volder, M. (2019). Hardware sequencing of inflatable nonlinear actuators for autonomous soft robots. Adv. Mater. ***31***, 1804598.
27. Lin, Y., Xu, Y.X., and Juang, J.Y. (2023). Single-actuator soft robot for in-pipe crawling. Soft Robot. ***10***, 174–186.
28. Deng, B., Chen, L., Wei, D., Tournat, V., and Bertoldi, K. (2020). Pulse-driven robot: motion via solitary waves. Sci. Adv. ***6***, eaaz1166.
29. Vasios, N., Gross, A.J., Soifer, S., et al. (2020). Harnessing viscous flow to simplify the actuation of fluidic soft robots. Soft Robot. ***7***, 1–9.
30. Paez-Granados, D., Yamamoto, T., Kadone, H., et al. (2021). Passive flow control for series inflatable actuators: application on a wearable soft-robot for posture assistance. IEEE Robot. Autom. Lett. ***6***, 4891–4898.
31. Cai, M., Jiao, Z., Nie, S., Wang, C., Zou, J., and Song, J. (2021). A multifunctional electronic skin based on patterned metal films for tactile sensing with a broad linear response range. Sci. Adv. ***7***, eabl8313.
32. Jiao, Z., Ye, Z., Zhu, P., Tang, W., Yang, H., and Zou, J. (2023). Self-sensing actuators with programmable actuation performances for soft robots. Sci. China Technol. Sci. ***66***, 3070–3079.
33. Shen, Z., Zhu, X., Majidi, C., et al. (2021). Cutaneous ionogel mechanoreceptors for soft machines, physiological sensing, and amputee prostheses. Adv. Mater. ***33***, 2102069.
34. Zhao, H., O Brien, K., Li, S., et al. (2016). Optoelectronically innervated soft prosthetic hand via stretchable optical waveguides. Sci. Robot. ***1***, eaai7529.
35. Hellebrekers, T., Kroemer, O., and Majidi, C. (2019). Soft magnetic skin for continuous deformation sensing. Adv. Intell. Syst. ***1***, 1900025.
36. Li, Z., Kidambi, N., Wang, L., et al. (2020). Uncovering rotational multifunctionalities of coupled kresling modular structures. Extreme Mech. Lett. ***39***, 100795.
37. Zhai, Z., Wang, Y., and Jiang, H. (2018). Origami-inspired, on-demand deployable and collapsible mechanical metamaterials with tunable stiffness. Proc. Natl. Acad. Sci. USA ***115***, 2032–2037.
38. Pagano, A., Yan, T., Chien, B., Wissa, A., and Tawfick, S. (2017). A crawling robot driven by multi-stable origami. Smart Mater. Struct. ***26***, 094007.
39. Rothemund, P., Ainla, A., Belding, L., Preston, D.J., Kurihara, S., Suo, Z., and Whitesides, G.M. (2018). A soft, bistable valve for autonomous control of soft actuators. Sci. Robot. ***3***, eaar7986.
40. Preston, D.J., Rothemund, P., Jiang, H.J., Nemitz, M.P., Rawson, J., Suo, Z., and Whitesides, G.M. (2019). Digital logic for soft devices. Proc. Natl. Acad. Sci. USA ***116***, 7750–7759.

41. Rajappan, A., Jumet, B., Shveda, R.A., Decker, C.J., Liu, Z., Yap, T.F., Sanchez, V., and Preston, D.J. (2022). Logic-enabled textiles. Proc. Natl. Acad. Sci. USA ***119***, e2202118119.
42. Tracz, J.A., Wille, L., Pathiraja, D., Kendre, S.V., Pfisterer, R., Turett, E., Abrahamsson, C.K., Root, S.E., Lee, W.K., Preston, D.J., Jiang, H.J., Whitesides, G.M., and Nemitz, M.P. (2022). Tube-balloon logic for the exploration of fluidic control elements. IEEE Robot. Autom. Lett. ***7***, 5483–5488.
43. Decker, C.J., Jiang, H.J., Nemitz, M.P., Root, S.E., Rajappan, A., Alvarez, J.T., Tracz, J., Wille, L., Preston, D.J., and Whitesides, G.M. (2022). Programmable soft valves for digital and analog control. Proc. Natl. Acad. Sci. USA ***119***, e2205922119.
44. Lu, H., Zhang, M., Yang, Y., Huang, Q., Fukuda, T., Wang, Z., and Shen, Y. (2018). A bioinspired multilegged soft millirobot that functions in both dry and wet conditions. Nat. Commun. ***9***, 3944.
45. Mao, Z., Iizuka, T., and Maeda, S. (2021). Bidirectional electrohydrodynamic pump with high symmetrical performance and its application to a tube actuator. Sens. Actuator A Phys. ***332***, 113168.
46. Tang, W., Zhang, C., Zhong, Y., Zhu, P., Hu, Y., Jiao, Z., Wei, X., Lu, G., Wang, J., Liang, Y., Lin, Y., Wang, W., Yang, H., and Zou, J. (2021). Customizing a self-healing soft pump for robot. Nat. Commun. ***12***, 2247.
47. Chen, R., Yuan, Z., Guo, J., Bai, L., Zhu, X., Liu, F., Pu, H., Xin, L., Peng, Y., Luo, J., Wen, L., and Sun, Y. (2021). Legless soft robots capable of rapid, continuous, and steered jumping. Nat. Commun. ***12***, 7028.
48. Diteesawat, R.S., Helps, T., Taghavi, M., et al. (2021). Electro-pneumatic pumps for soft robotics. Sci. Robot. ***6***, eabc3721.
49. Onal, C.D., Chen, X., Whitesides, G.M., et al. (2017). Soft mobile robots with on-board chemical pressure generation. In The 15th International Symposium ISRR (Springer International Publishing), pp. 525–540.

ACKNOWLEDGMENTS

This work was financially supported by the Zhejiang Provincial Natural Science Foundation of China (grant no. LD22E050002), the National Natural Science Foundation of China (grant no. 52205073), the China National Postdoctoral Program for Innovative Talents (grant no. BX2021258), and the China Postdoctoral Science Foundation (grant no. 2022M710125).

AUTHOR CONTRIBUTIONS

Z.J. and J.Z. conceived the idea and designed the experiments. Z.J., Z.H., Y.S., P.Z., W.T., and Y.Z. conducted the experimental work. Z.J. carried out the finite element simulation analysis, analyzed the data, and wrote the manuscript. J.Z. and H.Y. guided and supervised the whole project. Z.J., J.Z., K.X., F.L., and H.Y. revised the manuscript. All of the authors discussed the data and prepared the manuscript.

DECLARATION OF INTERESTS

The authors declare no competing interests.

SUPPLEMENTAL INFORMATION

It can be found online at https://doi.org/10.1016/j.xinn.2023.100549.

LEAD CONTACT WEBSITE

https://person.zju.edu.cn/0006327/786132.html.

The Innovation, Volume *5*

Supplemental Information

Reprogrammable, intelligent soft origami LEGO coupling actuation, computation, and sensing

Zhongdong Jiao, Zhenhan Hu, Yuhao Shi, Kaichen Xu, Fangye Lin, Pingan Zhu, Wei Tang, Yiding Zhong, Huayong Yang, and Jun Zou

The Innovation, Volume ■ ■

Supplemental Information

Reprogrammable, intelligent soft origami LEGO coupling actuation, computation, and sensing

Zhongdong Jiao, Zhenhan Hu, Yuhao Shi, Kaichen Xu, Fangye Lin, Pingan Zhu, Wei Tang, Yiding Zhong, Huayong Yang, and Jun Zou

Fabrication of the ReISOs

The ReISO can be readily fabricated through elastomer casting or LCD 3D printing. In the elastomer casting, all the molds are made of polylactic acid (PLA) and manufactured with a 3D printer (Trianglelab, Dforce 300). We used two kinds of elastomers: the chamber of the soft origami is made of E630 elastomer (Shenzhen Hong Ye Jie Technology Co., Ltd.), and the male and female connectors are made of E650 elastomer (Shenzhen Hong Ye Jie Technology Co., Ltd.). As illustrated in Figure S1, the fabrication process includes four steps: (1) Two components of elastomers and pigment were mixed with a glass rod and degassed in a vacuum container. (2) The elastomer mixtures were then poured into the molds and heated in an oven (DZF-6090AB, Lichen) at 65 °C for 30 min. (3) The cured elastomers were removed from the molds. (4) The elastomer components were stuck together using silicone adhesive (CT772B, Odake). In the LCD 3D printing, the ReISO is fabricated by curing flexible resin in a commercial 3D printer (LD-002H, CREALITY-3D), as shown in Figure S2. Then the control tube and intelligent tube are glued to the cured resin. The dimensions of the ReISOs are depicted in Figure S19.

The control system for the ReISOs

The ReISOs in the logic characterization experiments and the soft logic circuits are controlled by a custom-built pneumatic control system shown in Figure S13. The vacuum pressure is generated by a vacuum pump (V-I240SV pump, VALUE) and regulated with a pressure regulator (ITV 2090, SMC). The control signals for the pressure regulator are generated by a signal generator. The solenoid valve (KVE32PL24FF valve, Kamoer) is used to fold/unfold the ReISOs. The two inlet ports and the outlet port are connected to the vacuum, atmospheric air, and soft origami, respectively. The operation states of the solenoid valve are controlled via the digital signals produced by an Arduino board (MEGA2560 R3) and relays. The pressures at port “A” and port “Q” are measured using a custom-built data acquisition system. The

internal diameter of the elastomer tubes used in the control system is 2 mm.

Finite element model for the soft origami

To guarantee reliable blocking behavior, the contraction of the soft origami must be larger than the contraction required to block the airflow completely. We investigated the influence of the structure parameters on the deformation performances of the soft origami by finite element method (FEM). The commercial package ABAQUS 2019 was used for the finite element analyses. The soft origami was modeled with a Yeoh hyperelastic model, where material parameters were determined experimentally by uniaxial tensile tests. In Figure S10, we report the numerically predicted deformation performances of soft origami as a function of the initial origami height H_0 and side length L_s. Combined with the results in Figure 2A-C, we can obtain a design guideline of the soft origami. In this work, we choose H_0 = 30 mm and L_s = 30 mm to carry out characterization experiments and build soft machines.

Analytical model of the ReISO

The fluidic soft origami can be equivalent to an electric circuit, as shown in Figure 2F-G. In this fluidic circuit, the fluidic pressure (Pa), mass flow rate (kg/s), fluidic resistance (Pa • s/kg), and fluidic capacity (kg/Pa) are analogous to the voltage (V), current (A), resistance (Ω), and capacity (F) of electric circuit, respectively. R_{tube1}, R_{tube2}, and R_{capi} represent the fluidic resistance of the control tube, the upper part of the intelligent tube, and the capillary tube, respectively. C_{cham} and C_Q denote the fluidic capacities of the chamber of the soft origami and the airtight channel that connects with port "Q", respectively. P_{cham} and P_Q are the pressure of the chamber and the airtight channel, respectively. S_A is a mechanical switch that represents the state of input "A", and S_K is a fluidic relay that represents the state of the intelligent tube. When P_{cham} > P_{kink}, the relay SK is in state 1 (Figure 2F) where port "Q" is subjected to the vacuum. When $P_{cham} \leqslant P_{kink}$, the relay SK switches to state 2 (Figure 2G) where port "Q" is

connected to the atmosphere.

The Reynolds number in the system is approximately 10-100, which is significantly smaller than the critical Reynolds number (Re ~ 2,300) for the transition to turbulent flow. Then the fluidic resistance R_{fluid} can be calculated according to the Darcy-Weisbach equation for laminar flow:

$$R_{fluid} = \frac{\Delta P}{\dot{m}} = \frac{128\mu L}{\pi\rho D^4} \tag{1}$$

where ΔP is the pressure difference between the two ends of the fluidic tube, $\dot{m}$ is the mass flow rate of air, μ is the dynamic viscosity of air, ρ is the density of air, D is the internal diameter of the tube, and L is the length of the tube.

The fluidic capacity can be calculated with the ideal gas equation of state:

$$C_{fluid} = \frac{dm}{dP} = \frac{VM}{RT} \tag{2}$$

where V is the volume of the chamber or tube, M is the molar mass of air, R is the universal gas constant, and T is the temperature.

In the folding process, the air flows from the chamber to the vacuum source via R_{tube1}. When the pressure of the chamber P_{cham} reaches P_{kink} (the kinking pressure of the ReISO), the air flows from the atmosphere to the airtight channel that connects with port "Q" via R_{capi}. The pressures of the chamber and the intelligent tube can be described as:

$$P_S = C_{cham}\frac{dP_{cham}}{dt}R_{tube1} + P_{cham} \tag{3}$$

$$R_{capi}C_Q\frac{dP_Q}{dt} + P_Q = 0 \tag{4}$$

With the condition $t = 0$, $P_{cham} = P_{atm}$; $t = t_{kink}$, $P_Q = P_{Q0}$, the solution to Eq. 4-5 is:

$$P_{cham} = \frac{P_S}{R_{tube1}C_{cham}} + e^{-\frac{1}{R_{tube1}C_{cham}}t}\left(P_{atm} - \frac{P_S}{R_{tube1}C_{cham}}\right) \tag{5}$$

$$P_Q = \begin{cases} P_{Q0} & t < t_{kink} \\ P_{Q0}e^{-\frac{1}{R_{capi}C_Q}(t-t_{kink})} & t \geq t_{kink} \end{cases} \tag{6}$$

$$P_{Q0} = \frac{R_{capi}}{R_{tube2} + R_{capi}} P_S \tag{7}$$

$$t_{kink} = -R_{tube1} C_{cham} \ln \frac{R_{tube1} C_{cham} P_{kink} - P_S}{R_{tube1} C_{cham} P_{atm} - P_S} \tag{8}$$

where P_{Q0} is the pressure of port "Q" in the unfolding state.

In the unfolding process, the air flows into the chamber via R_{tube1}. When the internal pressure of the chamber is higher than P_{kink}, the intelligent tube is open and the air at the "Q" port is removed.

$$R_{tube1} C_{cham} \frac{dP_{cham}}{dt} + P_{cham} = 0 \tag{9}$$

$$\left(\frac{P_Q}{R_{capi}} + C_Q \frac{dP_Q}{dt} \right) R_{tube2} + P_Q = P_S \tag{10}$$

With the condition $t = 0$, $P_{cham} = P_S$; $t = t_{open}$, $P_Q = P_{atm}$, the solution to Eq. 10-11 is:

$$P_{cham} = P_S e^{-\frac{1}{R_{tube1} C_{cham}} t} \tag{11}$$

$$P_Q = \begin{cases} P_{atm} & t < t_{open} \\ X_2 + e^{-X_1 \left(t - t_{open} \right)} \left(P_{atm} - X_2 \right) & t \geq t_{open} \end{cases} \tag{12}$$

$$X_1 = \frac{R_{tube2} + R_{capi}}{R_{tube2} R_{capi} C_Q}, \quad X_2 = \frac{P_S}{R_{tube2} C_Q}$$

$$t_{open} = -R_{tube1} C_{cham} \ln \frac{P_{open}}{P_S} \tag{13}$$

The logic response time (the time required to switch from Q = "1" to Q = "0" or from Q = "0" to Q = "1") of the ReISO can be expressed as Eq. 15 and Eq. 17

$$t_{1 \to 0} = -R_{capi} C_Q \ln \frac{P_{Q1}}{P_{Q0}} + t_{kink} \tag{14}$$

$$P_{Q1} = \left(P_{Q0} - P_{atm} \right) \times 10\% + P_{atm} \tag{15}$$

$$t_{0 \to 1} = -\frac{1}{X_1} \ln \frac{P_{Q2} - X_2}{P_{atm} - X_2} + t_{open} \tag{16}$$

$$P_{Q2} = \left(P_{Q0} - P_{atm} \right) \times 90\% + P_{atm} \tag{17}$$

The state that $P_Q \geqslant P_{Q1}$ is defined as the logic low state, and the state that $P_Q \leqslant P_{Q2}$ is defined as the logic high state (here the fluidic signals processed by the ReISO are vacuum pressures, thus the magnitude of P_Q, P_{Q1}, and P_{Q2} is negative).

For the ReISOs in this work, we choose the elastomer tubes with an internal diameter of 2.0 mm and an external diameter of 3.0 mm as the input tubes and intelligent tubes owing to their superior kinking properties. The tubes with this dimension allow the ReISOs to exhibit a relatively fast response (~ 0.2 s), thus, $t_{0\to1}$ is very small (< 1 s). In contrast, the capillary tubes have high fluidic resistance and can be utilized to modulate the response characterization of the ReISOs. Then we employed this analytical model to calculate the $t_{1\to0}$ of the ReISOs. The results are shown in Figure 2I, in which the calculated response time agrees well with the experimental values. The agreement between the model and experiment illustrates the potential of analyzing complex soft circuits constructed with ReISOs.

The frequency characteristic and fatigue test of the ReISOs

We further studied the logic performances of ReISO under frequencies between 0.1 and 2.5 Hz. The highest frequency that allowed for the logic operation was 2.5 Hz (Figure S8F). The result of a continuous fatigue test of the soft origami sample over 10,000 operating cycles under a pressure of -80 kPa and a frequency of 2.5 Hz is shown in Figure S9. The soft origami consistently exhibited the same output pressure and logic operation capability.

The working principle of the soft turtle

As the soft turtle swims in the tank, its legs swing periodically in the water (Figure 5D). The swinging movement makes the legs be subjected to a reaction torque, which can be calculated with the drag equation.

$$F_d = \frac{1}{2}\rho v^2 C_d A \tag{18}$$

$$M_d = F_d L = \frac{1}{2}\rho v^2 C_d A L_d \tag{19}$$

where F_d is the drag force, ρ is the density of water, v is the moving velocity of the legs relative to the water, C_d is the drag coefficient, A is the projected area of the legs in the moving direction, L_d is the equivalent distance between the turtle leg and the axis of the soft origami, M_d is the drag torque.

If the swinging speeds are the same in the folding and unfolding processes, the turtle will stay put. However, for the ring oscillator, the soft origami is folded via the intelligent tube whose internal diameter is 2 mm, and unfolded via the capillary tube whose internal diameter is 0.7 mm. The differences in the internal diameter lead to different flow rates, therefore causing the morphing speed differences between folding and unfolding processes (Figure 5D). As a result, the turtle moves toward the thrust direction of the folding process.

$$W = \int_{\theta_1}^{\theta_2} M_d d\theta = \frac{1}{2}\rho v^2 C_d A L_d d\theta \tag{20}$$

$$v_{fold} > v_{unfold} \tag{21}$$

$$W_{fold} > W_{unfold} \tag{22}$$

where W is the amount of the work, W_{fold} and W_{unfold} are the amounts of the work in the folding and unfolding processes, respectively. v_{fold} and v_{unfold} are the moving velocity of the legs in the folding and unfolding processes, respectively. θ is the twisting angle of the leg.

The swimming speed of the soft turtle can be modulated by tuning the oscillation frequency and range (twisting angle) of the soft ring oscillator. These properties are determined by two key parameters: the actuation pressure and the internal diameter of the capillary tube. As illustrated in Figures S18A and S18C, an increase in the internal diameter of the capillary tube results in higher oscillation frequency, while having a negligible influence on the oscillation range. Conversely, the oscillation range first

increases with the decrease in vacuum pressure, but then remains at a certain level (actuation pressure < -40 kPa). The oscillation frequency has slight variations with varying vacuum pressure (Figures S18B and S18D).

Characterization experiments of the ReISOs

The experimental setup for actuation performance tests is demonstrated in Figure S11. Two high-resolution cameras are placed on top of the module and in front of the module, respectively. The angle and height variations of the module are recorded by the camera at a frame rate of 30 fps.

The setup for kinking characterization of the elastomer tubes is illustrated in Figure S12. One end of the tube is fixed, while the other end of the tube is driven by two stepping motors. Stepping motor 1 is responsible for twisting the tube to a certain angle, and stepping motor 2 is used to compress the tube.

The working principle of the soft ring oscillator

When the source ports of the three ReISOs were connected to a constant vacuum pressure, module "A" was actuated first (the module that is actuated first is random). As described in Figure S15A, the deformation of module "A" blocked the input port of module "B" in S1. Subsequently, module "C" was evacuated via the output port of module "B". When module "C" became the logic high state (the internal intelligent tube was kinked), the ring oscillator came into state S2 (Figure S15B), with the input port of module "A" being blocked and module "A" returning to its original shape. The opening of the airflow in module "A" made module "B" be subjected to vacuum pressure. When the internal pressure of module "B" reached the kinking pressure, the input port of module "C" is blocked and module "C" returned to the logic low state gradually (state S3, Figure S15C). The reopening of the airflow of module "C" initiated the actuation of module "A", and the soft ring oscillator entered the next cycle.

The JK flip flop and D flip flop circuits enabled by the ReISOs

JK Flip Flop

The JK flip flop is a gated SR latch with the addition of a clock input that prevents the illegal or invalid output when both inputs “S” and “R” are set to “1” (Figure S16A-i). In this circuit, the inputs “S” and “R” are labeled as “J” and “K”, respectively. The two input ports are replaced by two 3-input NAND gates with the second input of each gate connected to a clock input and the third input connected to the output “Q” or “$\overline{Q}$”. This feedback enables only one of its two input ports, either SET or RESET to be active at any time under normal switching. Therefore, the invalid condition of “J” = “1” and “K” = “1” state can be used to produce a “toggle action” as the two inputs are now interlocked.

In this work, ten ReISOs were employed to construct a soft JK flip flop circuit, which is positive edge-triggered, as depicted in Figure S16A-ii. When both inputs “J” and “K” were “0”, the outputs held the previous state. If “J” was set to “1” and “K” was set to “0”, the rising edge of the clock input caused the output “Q” to become “1” and “$\overline{Q}$” to become “0” (Figure S16A-iii-iv and Movie S8). Similarly, when “J” was set to “0” and “K” was set to “1”, the rising edge of the clock input caused the output “Q” to become “0” and “$\overline{Q}$” to become “1”. When both “J” and “K” were set to “1”, the output “Q” and “$\overline{Q}$” toggled the previous state with each rising edge of the clock input.

D Flip Flop

The D flip flop (delay flip flop) is designed by adding a NOT gate between the inputs “S” and “R” of a gated SR latch (Figure S16B-i). Then inputs “S” and “R” become complements of each other and are never equal to each other at the same time, allowing us to control the toggle action of the flip flop using one single input. The D flip flop has two inputs: the data input “D” and the clock input “Clk”. In this circuit, the data input is delayed up to one clock pulse before it is displayed in the output. Therefore, the D flip flop can be used to store data at a predetermined time and hold it until it is needed.

As illustrated in Figure S16B-ii, a soft D flip flop was created using 9 ReISOs. When the clock input was set to “0”, the outputs “Q” and “$\overline{Q}$” were held in the previous state. In contrast, the high level of the clock input enabled the input “D” to be copied to the output “Q” (Figure S16B-iii-iv and Movie S8).

The soft frequency enabled by the ReISOs

A soft frequency divider is based on a D flip flop circuit with the output "$\overline{\mathrm{Q}}$" connected directly back to the data input "D" (Figure 4G-i). This feedback ensures the output pulses at "Q" have a frequency that is exactly one half of the input clock frequency. As illustrated in Figure 4G-ii, the soft frequency divider was created using 9 ReISOs. It can be seen that the output "Q" only changes state on the rising edge of the pulse clock stream "Clk" (Figure 4G-iii-iv). Each rising edge occurs once every cycle, but the output "Q" requires two changes to complete a cycle. Therefore, the output "Q" changes at half the rate of the pulse clock stream "Clk". In other words, the clock frequency was divided by two. The frequency can be further divided by cascading more D flip flops. This demonstration illustrates that the ReISOs are able to process fluidic signals.

The vacuum leakage rate of the ReISOs

The vacuum leakage has some influences on the pressure magnitude of the vacuum system. As demonstrated in Figure S21, the vacuum leakage rate increases with the increase of the internal diameter of the capillary tube and the quantity of the ReISOs. When eight ReISOs with a capillary tube of 0.5 mm are connected in parallel, the vacuum system can retain 80.4% of the initial vacuum pressure (-56.3 kPa), which is sufficient to power soft actuators.

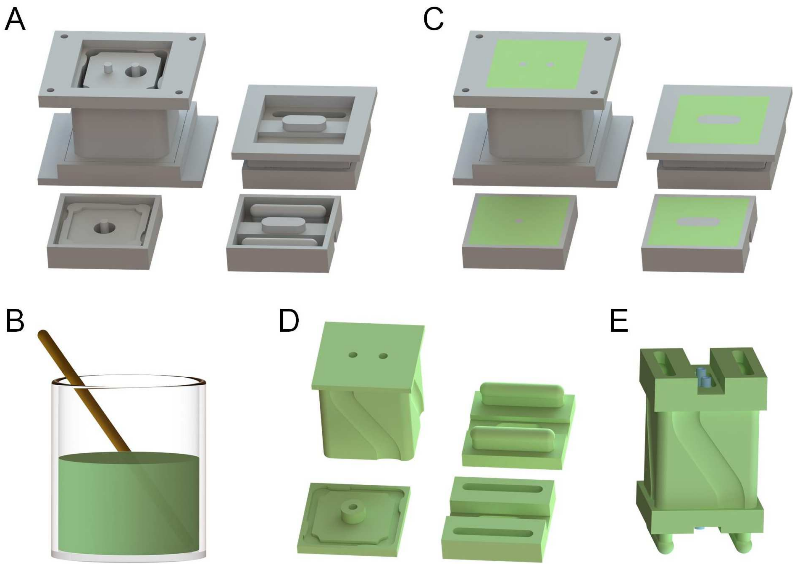

Figure S1. The ReISO is fabricated by elastomer casting. a All the molds are manufactured with a 3D printer. **b** The elastomer and pigment are mixed, stirred with a glass rod, and degassed in a vacuum container. **c** The liquid elastomer is poured into the mold and heated in an oven. **d** The cured elastomers are removed from the molds. **e** All the parts are stuck together using silicone adhesive, forming a soft origami.

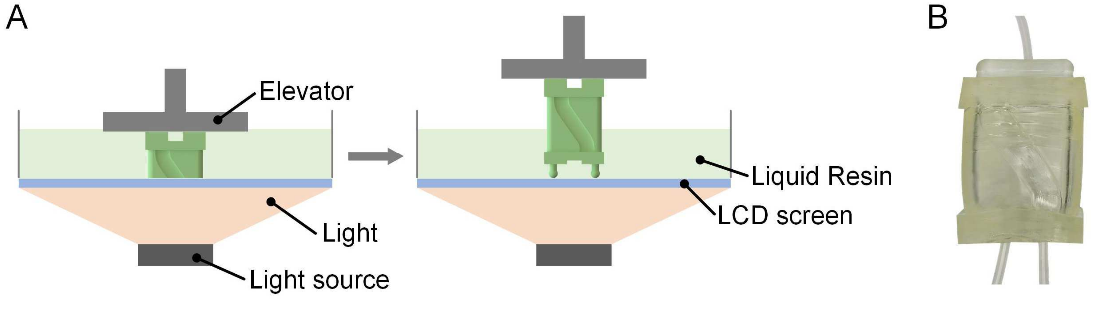

Figure S2. The ReISO is fabricated with an LCD printer. (A) The printing process of the ReISO. (B) The ReISO made of flexible resin.

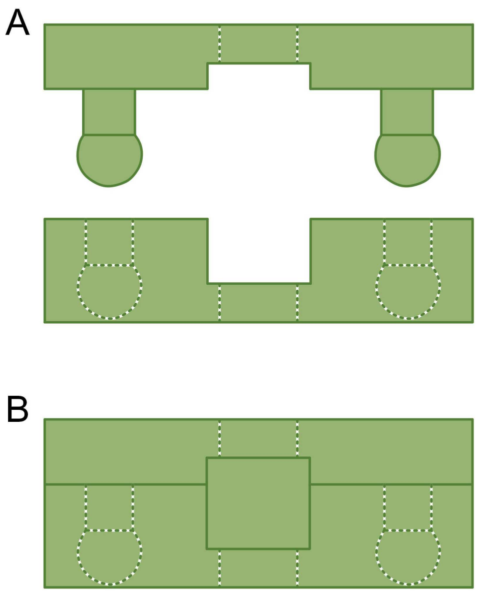

Figure S3. The plug-and-play connectors of the ReISOs. (A) Before assembly. (B) After assembly.

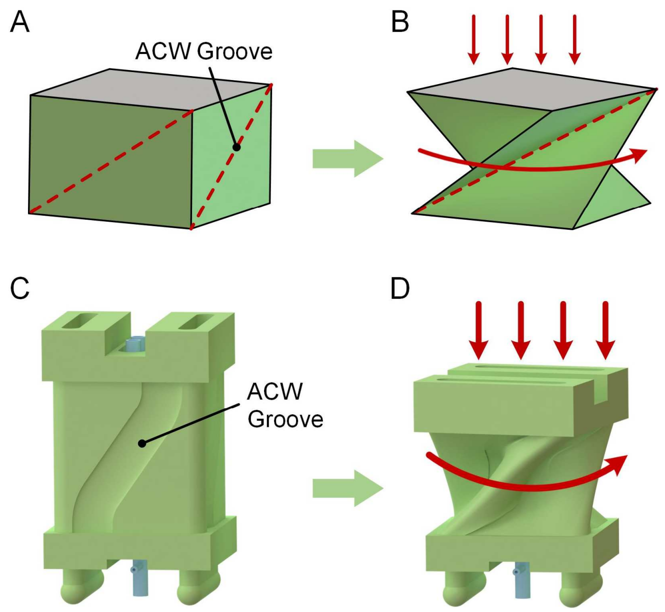

Figure S4. Schematic illustration of the Kresling origami and ReISO with anticlockwise grooves. (A-B) The anticlockwise Kresling origami is in unfolded (A) and folded (B) states. The red dashed lines represent the creases. The red arrows indicate the folding direction of the Kresling origami. ACW represents anticlockwise. (C-D) Schematic illustration of the anticlockwise ReISO in unfolded (C) and folded (D) states. The red arrows indicate the deformation direction of the soft origami. The grooves in the sides are the creases of the soft origami.

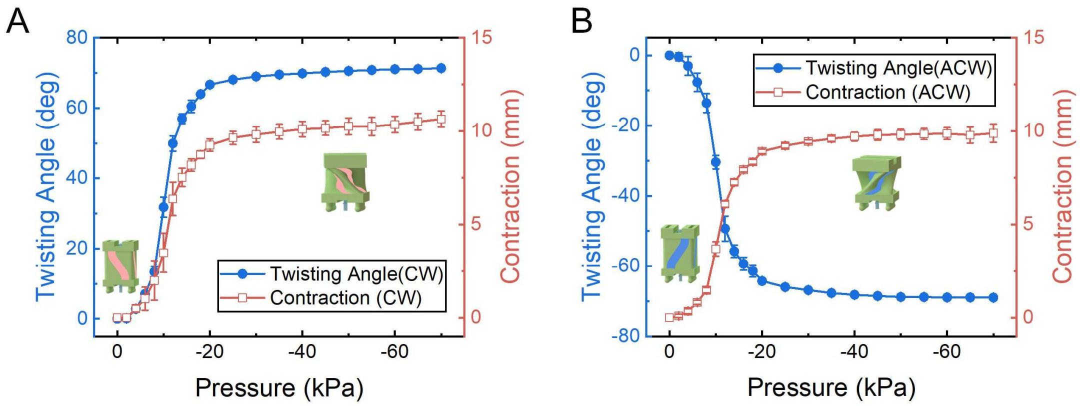

Figure S5. The twisting angle and contraction of the ReISOs with clockwise grooves (A) and anticlockwise grooves (B) at different vacuum pressures. CW represents clockwise, ACW represents anticlockwise.

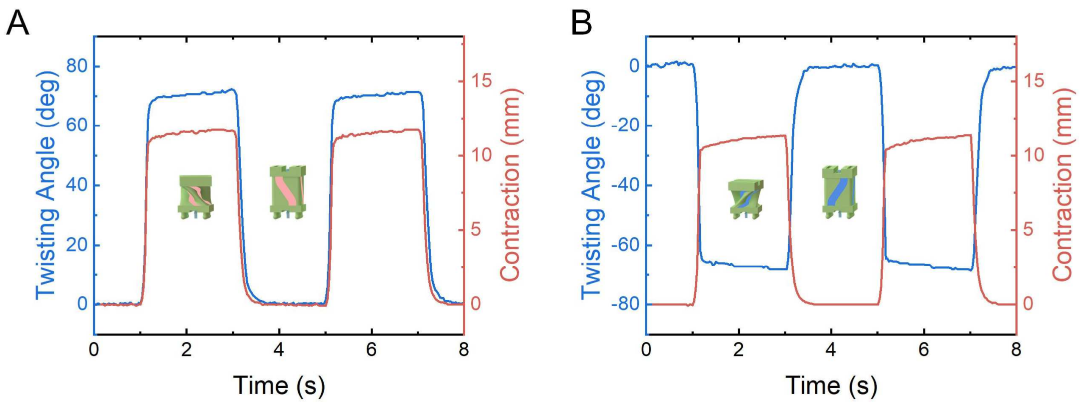

Figure S6. The dynamic deformation response of the ReISOs with clockwise grooves (A) and anticlockwise grooves (B).

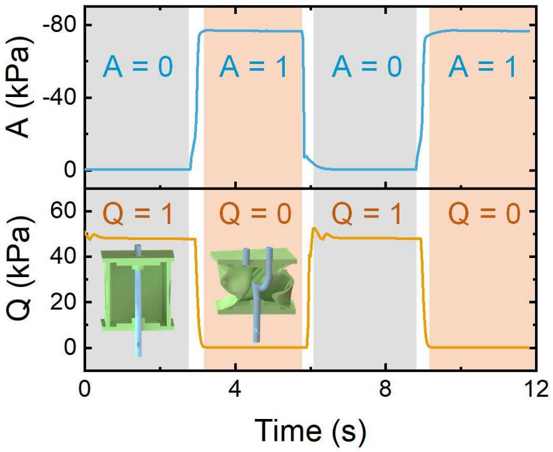

Figure S7. The pressure response of the ReISO when the "S" port is connected to a constant positive pressure of 60 kPa. In this case, the positive and vacuum pressure are defined as "1".

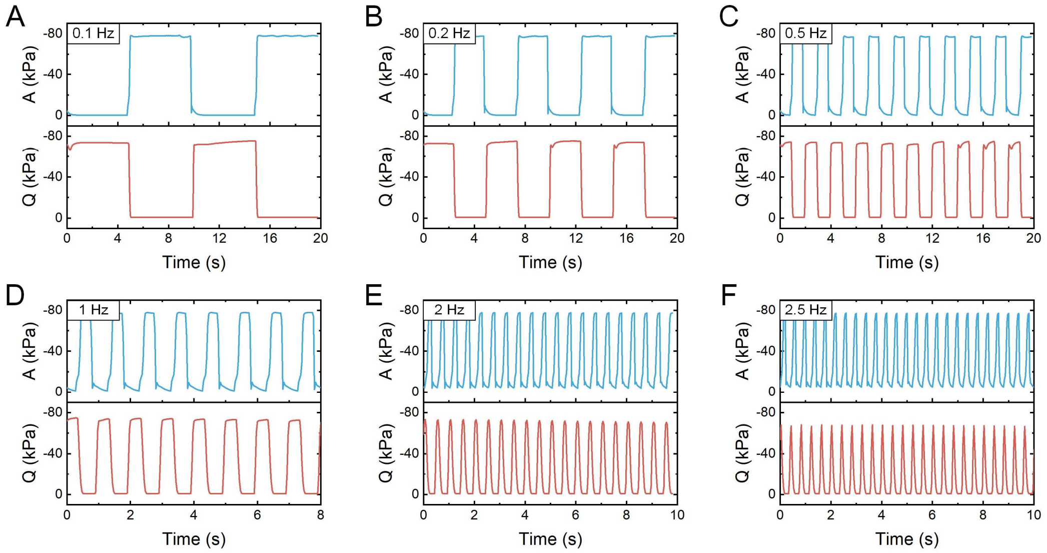

Figure S8. The pressure responses of the ReISO operating at different frequencies. (A) 0.1 Hz. (B) 0.2 Hz. (C) 0.5 Hz. (D) 1 Hz. (E) 2 Hz. (F) 2.5 Hz. The operation pressure is -80 kPa.

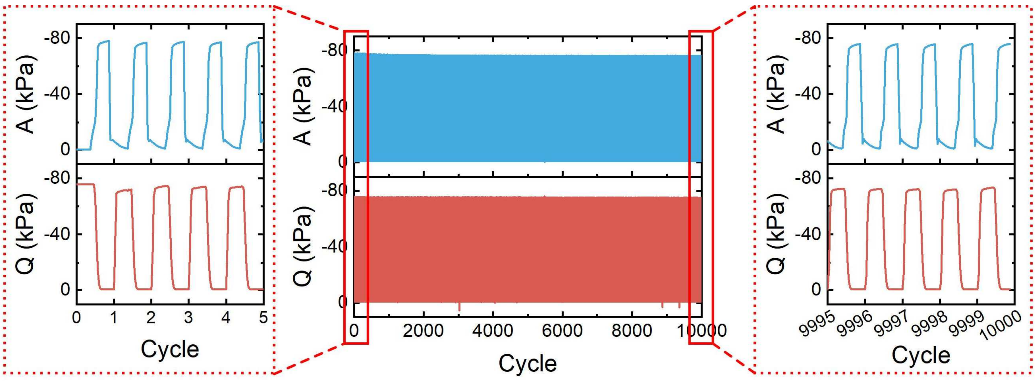

Figure S9. Fatigue test of the ReISO. A ReISO is actuated 10,000 cycles at a frequency of 2.5 Hz and a pressure of -80 kPa. The pressure response in the actuating process of the No. 1-5 and No. 9995-10000 cycles evidences the high durability of the ReISOs.

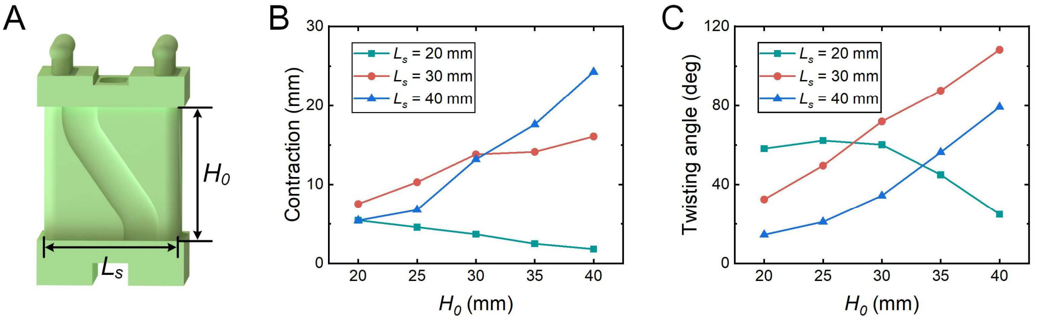

Figure S10. Finite element analysis of the soft origami with different dimensions. (A) The dimension schematic of the soft origami. H_0 is the initial origami height, L_s is the side length. (B) The contraction of the soft origami with different initial heights and side lengths. (C) The twisting angle of the soft origami with different initial heights and side lengths.

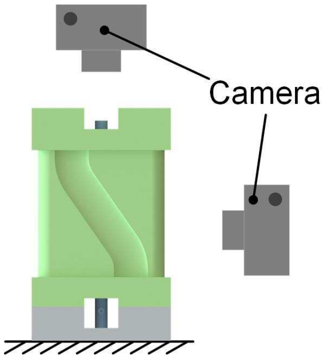

Figure S11. Experimental setup for the deformation measurement of the ReISOs.

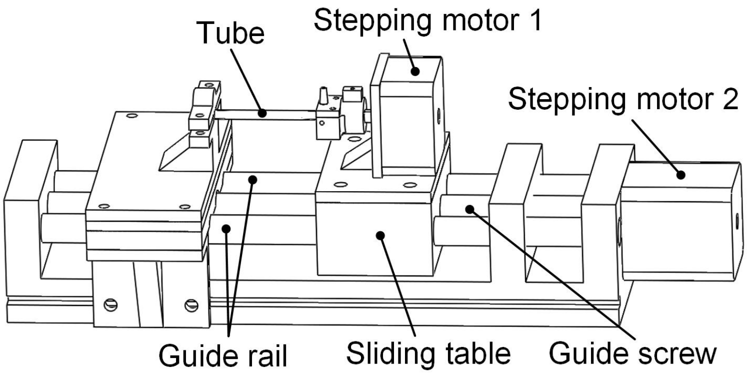

Figure S12. Experimental setup for the kinking characterization of the elastomer tubes.

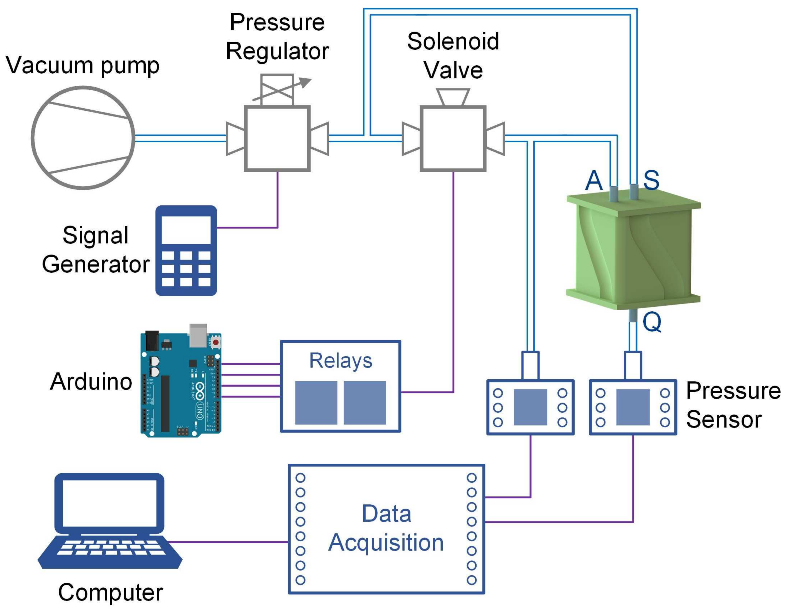

Figure S13. The schematic of the control system used to actuate the soft origami. The purple lines represent the control signals; the blue lines represent the fluidic channels.

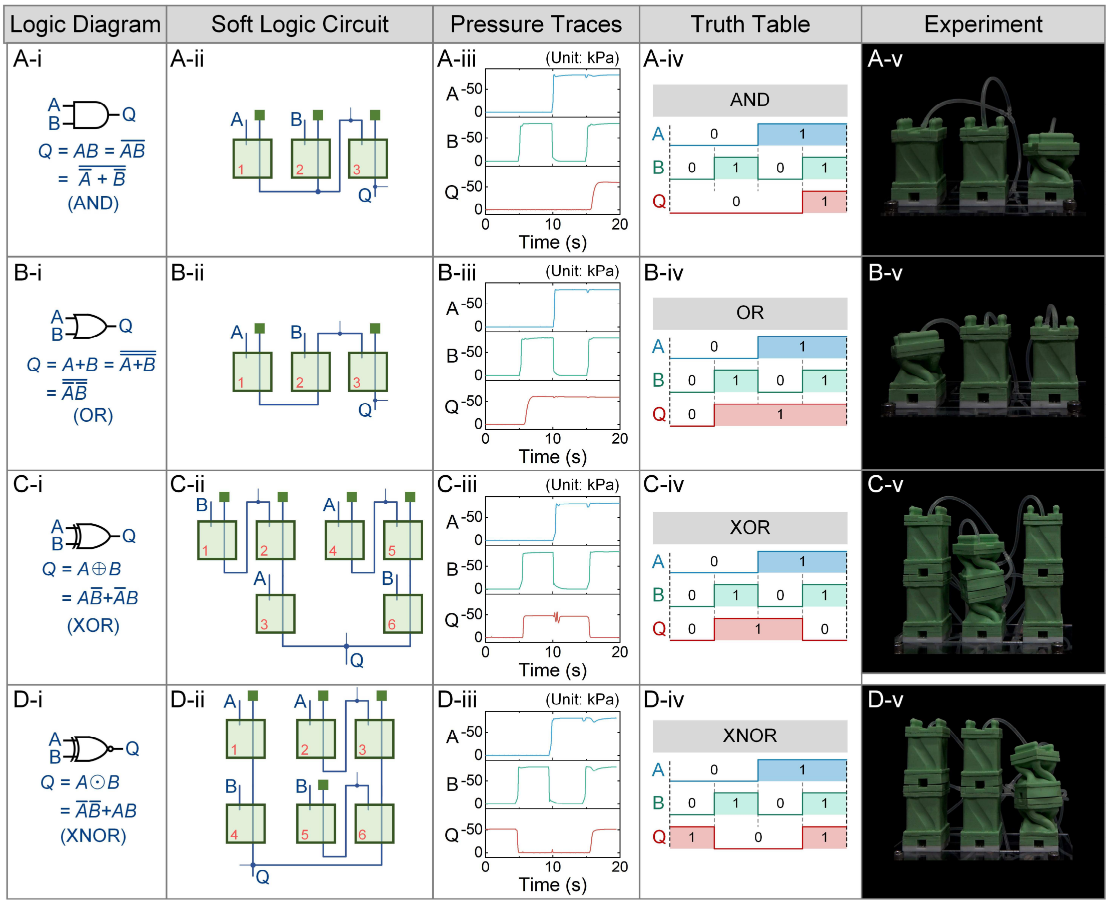

Figure S14. The reprogrammable combinatorial logic circuits based on the ReISOs. (A)-i The logic symbol and Boolean expression of the AND gate. (A)-ii The schematic of the soft AND gate circuit. (A)-iii The pressure traces of the soft AND gate. (A)-iv The truth table of the AND gate. (A)-v The experimental image of the soft AND gate. (B) The OR gate. (C) The XOR gate. (D) The XNOR gate. The green block represents the vacuum pressure.

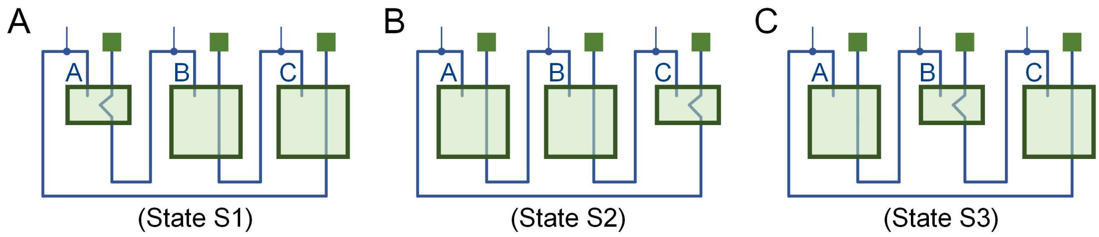

Figure S15. The oscillation sequences of the soft ring oscillator. Module “A” (A), module “C” (B), and module “B” (C) are folded sequentially.

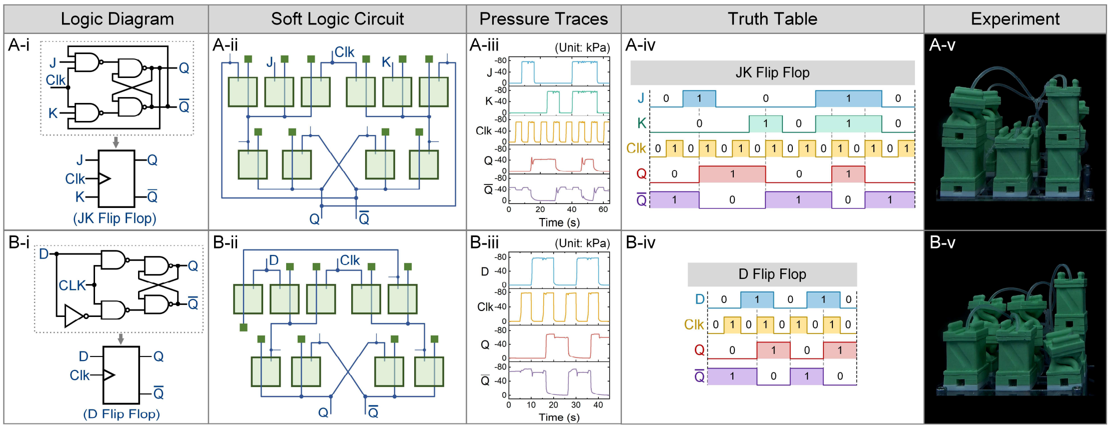

Figure S16. The reprogrammable sequential logic circuits based on the ReISOs. (A)-i The circuit diagram of the JK flip flop. (A)-ii The schematic of the soft JK flip flop circuit. (A)-iii The pressure traces of the soft JK flip flop. (A)-iv The truth table of the JK flip flop. (A)-v The experimental image of the soft JK flip flop. (B) The D flip flop. The green block represents the vacuum pressure.

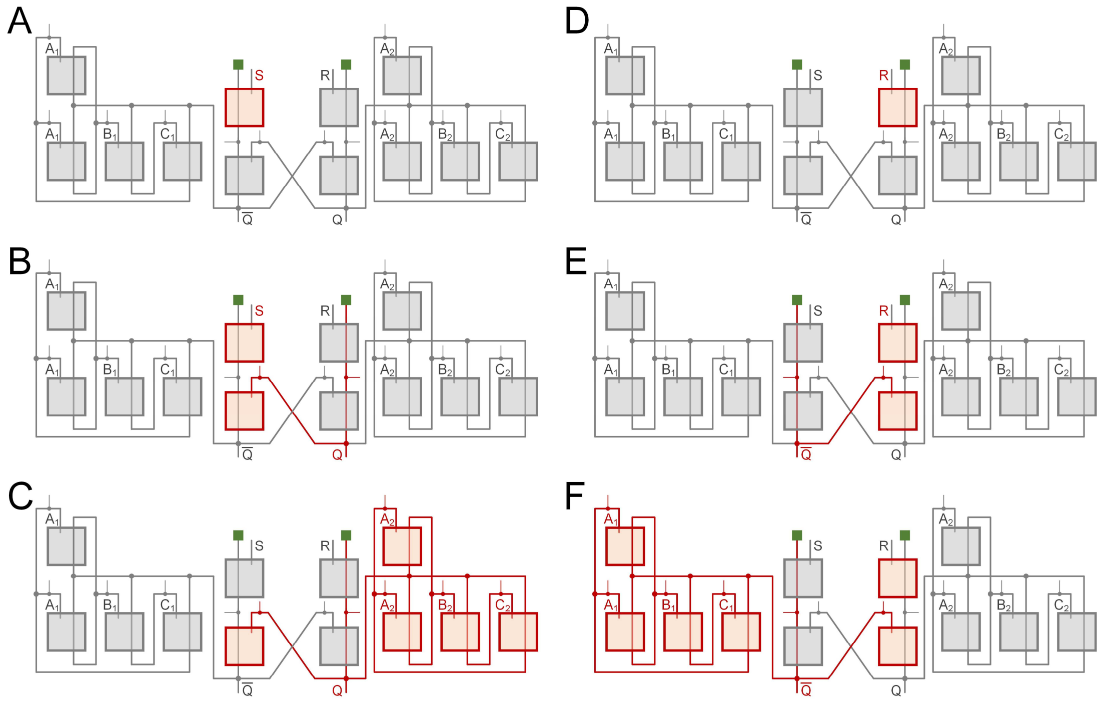

Figure S17. The control system of the untethered and autonomous soft turtle. (A) The twisting stimulus was applied to module “S”. (B) The soft SR latch circuit detected this stimulus and stored the current state (Q = “1”) in the circuit. (C) The Ring Oscillator-2 converted the constant pressure from port “Q” of the SR latch into oscillatory pressures, which drive the turtle to swim forward. The removal of the stimulus does not change the state of the SR latch circuit. (D) The twisting stimulus was applied to module “R”. (E) The soft SR latch circuit detected this stimulus and stored the current state ($\overline{Q}$ = “1”) in the circuit. (F) The Ring Oscillator-1 converted the constant pressure from port “$\overline{Q}$” of the SR latch into oscillatory pressures, which drive the turtle to swim backward.

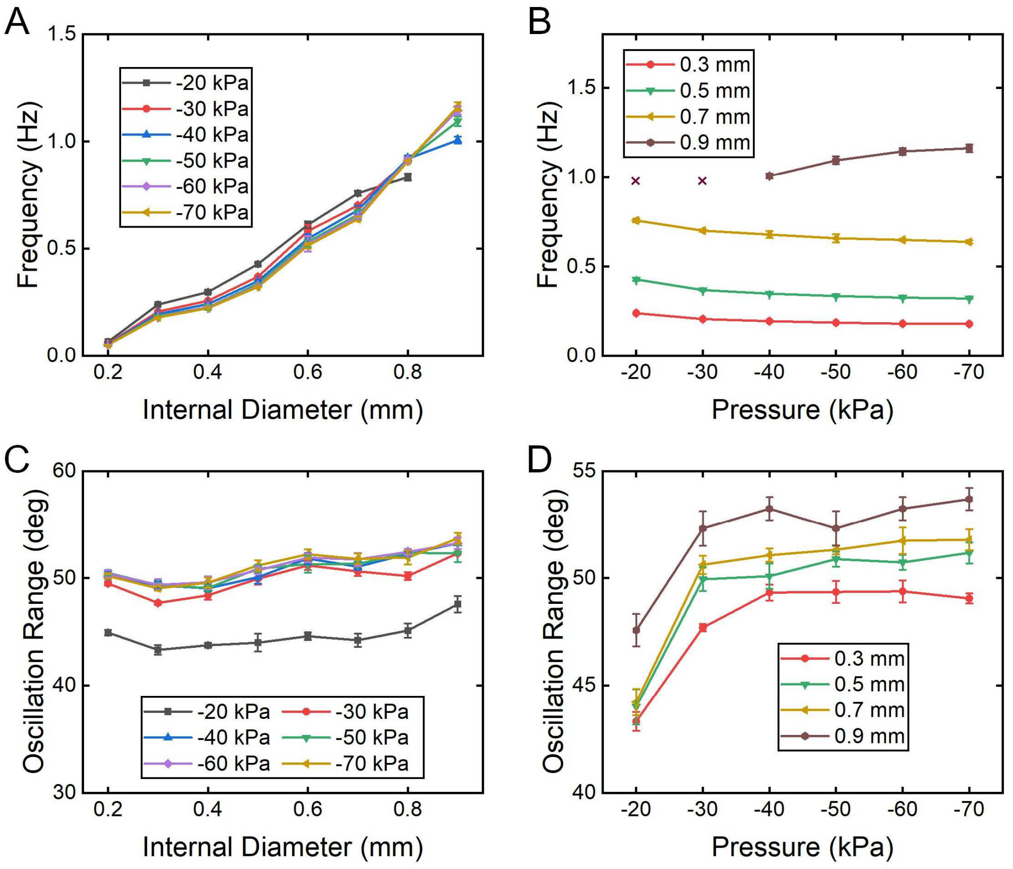

Figure S18. The oscillation frequency and range (twisting angle) can be adjusted by changing the inner diameter of the capillary tube and the actuation pressure of the ring oscillator. (A-B) Oscillation frequency as a function of the inner diameter of the capillary tube (A) and the actuation pressure (A). (C-D) Oscillation range as a function of the inner diameter of the capillary tube (C) and the actuation pressure (D).

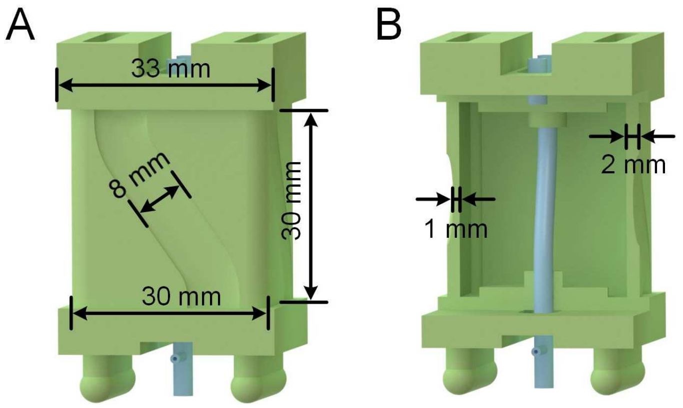

Figure S19. The dimension of the ReISO. (A) Front view. (B) cross-section view.

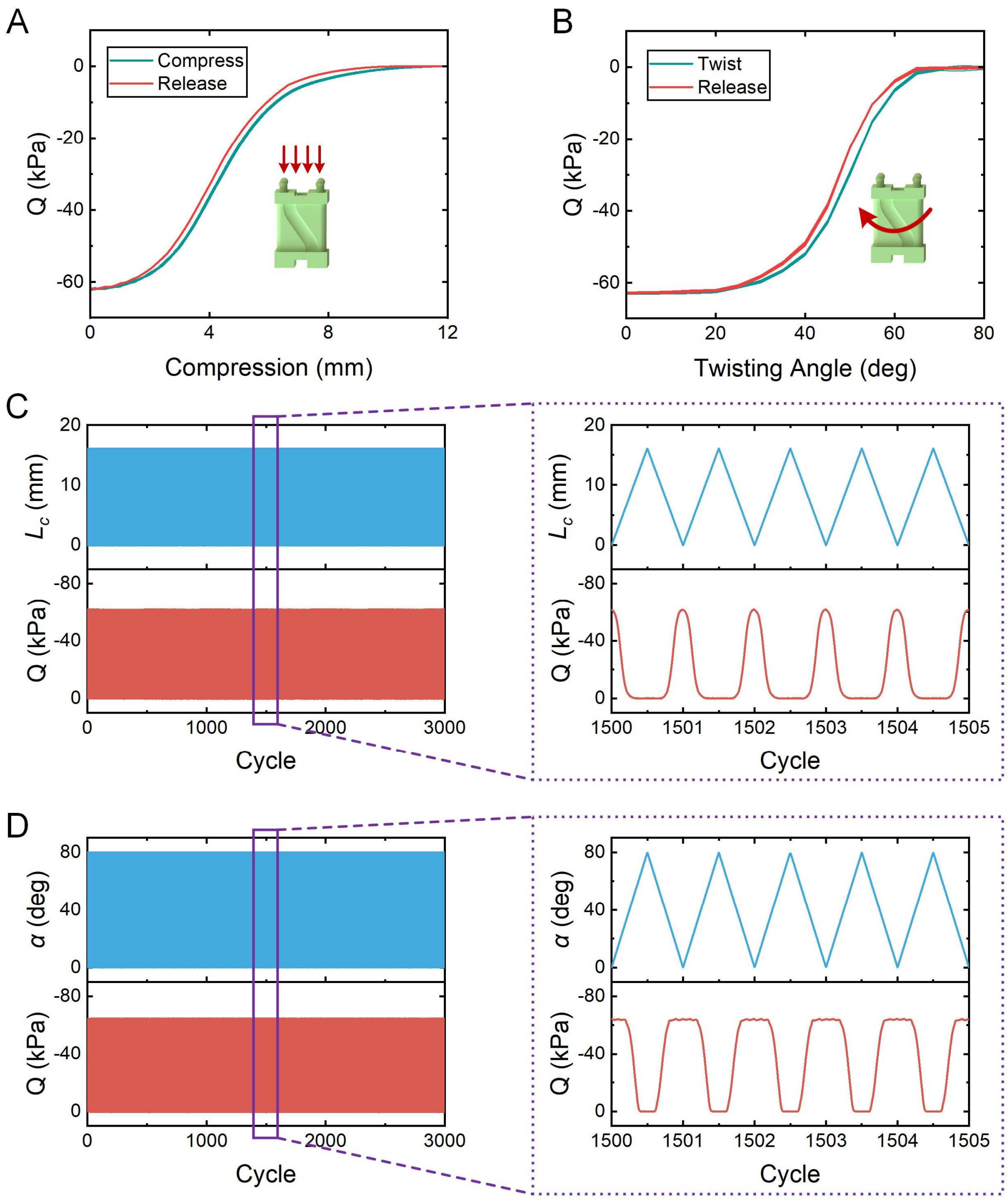

Figure S20. The sensing characteristic of the ReISOs. (A) The output pressure changes with varying compressions. The measurements were repeated five times. (B) The output pressure changes with varying twisting angles. (C) The fatigue test of the compression sensing capability of the ReISO. L_c represents compression. (D) The fatigue test of the twisting angle sensing capability of the ReISO. α represents twisting angle.

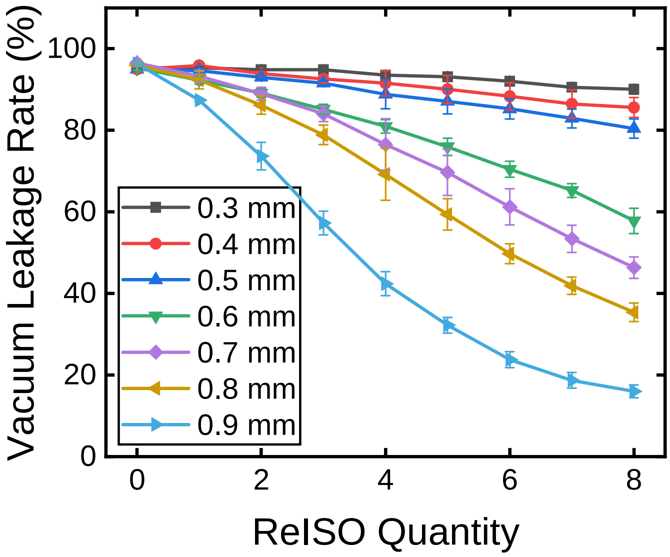

Figure S21. The vacuum leakage rate of the ReISOs with different capillary tube and module quantity.

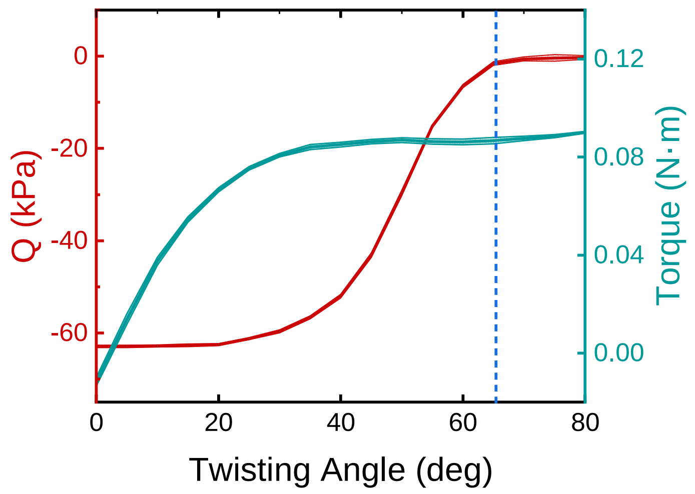

Figure S22. The torque and output pressure variations while twisting the ReISOs.

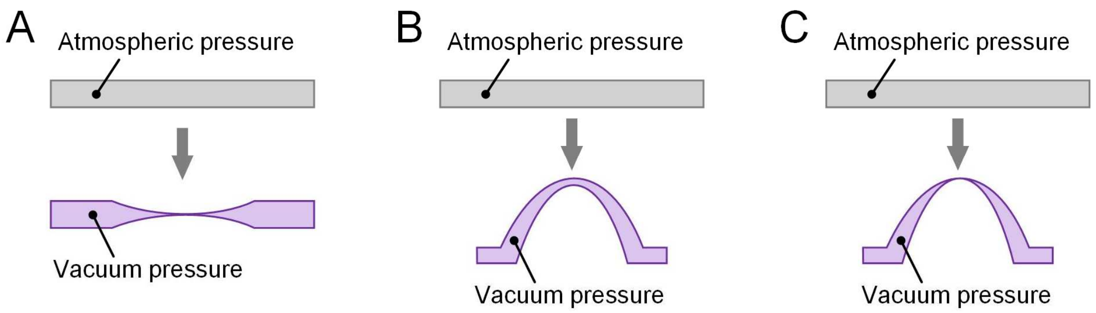

Figure S23. The deformation of the compressed elastomer tube. (A) The tube with a size of 1.5×2.0 mm collapses when subjected to vacuum pressure. (B) The tube with a size of 1.0×2.0 mm is difficult to be kinked due to smaller internal and external diameters. (C) The tube is successfully kinked.

Table S1. The Estimated cost of materials used for the fabrication of a ReISO.

Items	Amount	Unit	Cost
Elastomer	34	g	$ 0.693
Elastomer tube	0.2	m	$ 0.026
Silicone adhesive	2	mL	$ 0.133
Total cost			**$ 0.852**

Supplemental Movie Captions

Movie S1. The actuation capability of the ReISOs. The soft origami rotates clockwise and anticlockwise, respectively.

Movie S2. The logic operation capability of the ReISOs. Port “S” is connected to the pressurized air and vacuum, respectively. The vacuum/positive pressure and atmospheric pressure are defined as logic signal “1” and “0”, respectively.

Movie S3. The sensing capability of the ReISOs. The soft origami is able to detect twisting and pressing stimuli.

Movie S4. The damage-resistance of the ReISOs. The logic operation capability of the soft origami is not affected after being pricked with a needle.

Movie S5. The reconfigurable morphologies of the ReISOs. The ReISOs are configured into various morphologies to exhibit contraction movement, twisting movement, outward radial movement, inward radial movement, and bidirectional bending movement, respectively.

Movie S6. Reconfigurable soft robots based on the ReISOs. The soft rod-climbing robot is able to climb forward and backward along a metal rod. The soft manipulator is utilized to place triangular and square objects into holes with specific shapes.

Movie S7. Reprogrammable soft combinatorial logic circuits. The ReISOs are utilized to build fundamental combinatorial logic circuits, including the NOT gate, Buffer gate, NAND gate, NOR gate, AND gate, OR gate, XOR gate, and XNOR gate.

Movie S8. Reprogrammable soft sequential logic circuits. The ReISOs can be used to construct sequential logic circuits, including the ring oscillator, SR latch, JK flip flop, and D flip flop circuits.

Movie S9. Reprogrammable soft functional circuits. A soft full adder circuit and a soft frequency divider circuit are built by assembling the ReISOs.

Movie S10. The movements of an untethered soft turtle. The soft turtle is able to swim forward, swim backward, rotate clockwise, and rotate anticlockwise, respectively.

Movie S11. An untethered autonomous soft turtle that is able to sense stimuli, store data, process signals, and actuate muscles. The soft turtle can switch swimming gaits after sensing twisting stimuli.
